# *In silico/In vivo* analysis of high-risk papillomavirus L1 and L2 conserved sequences for development of cross-subtype prophylactic vaccine

**DOI:** 10.1038/s41598-019-51679-8

**Published:** 2019-10-23

**Authors:** Ali Namvar, Azam Bolhassani, Gholamreza Javadi, Zahra Noormohammadi

**Affiliations:** 1 Department of Biology, School of Basic Sciences, Science and Research Branch, Islamic Azad University, Tehran, Iran; 2 Department of Hepatitis and AIDS, Pasteur Institute of Iran, Tehran, Iran

**Keywords:** Predictive medicine, Viral infection

## Abstract

Human papillomavirus (HPV) is the most common sexually transmitted infection in the world and the main cause of cervical cancer. Nowadays, the virus-like particles (VLPs) based on L1 proteins have been considered as the best candidate for vaccine development against HPV infections. Two commercial HPV (Gardasil and Cervarix) are available. These HPV VLP vaccines induce genotype-limited protection. The major impediments such as economic barriers especially gaps in financing obstructed the optimal delivery of vaccines in developing countries. Thus, many efforts are underway to develop the next generation of vaccines against other types of high-risk HPV. In this study, we developed DNA constructs (based on L1 and L2 genes) that were potentially immunogenic and highly conserved among the high-risk HPV types. The framework of analysis include (1) B-cell epitope mapping, (2) T-cell epitope mapping (*i.e*., CD4^+^ and CD8^+^ T cells), (3) allergenicity assessment, (4) tap transport and proteasomal cleavage, (5) population coverage, (6) global and template-based docking, and (7) data collection, analysis, and design of the L1 and L2 DNA constructs. Our data indicated the 8-epitope candidates for helper T-cell and CTL in L1 and L2 sequences. For the L1 and L2 constructs, combination of these peptides in a single universal vaccine could involve all world population by the rate of 95.55% and 96.33%, respectively. *In vitro* studies showed high expression rates of multiepitope L1 (~57.86%) and L2 (~68.42%) DNA constructs in HEK-293T cells. Moreover, *in vivo* studies indicated that the combination of L1 and L2 DNA constructs without any adjuvant or delivery system induced effective immune responses, and protected mice against C3 tumor cells (the percentage of tumor-free mice: ~66.67%). Thus, the designed L1 and L2 DNA constructs would represent promising applications for HPV vaccine development.

## Introduction

Human papillomavirus (HPV) is the most common sexually transmitted infection in the world and the main cause of cervical cancer. Globally, 4.5% of all cancers worldwide (60,000 cases per year in men and 570,000 cases per year in women) are attributable to HPV^1^. More than 150 viral types of HPV have been identified whose classification is based on their association with cervical cancer and precursor lesions. HPV types were classified as high-risk (16, 18, 31, 33, 35, 39, 45, 51, 52, 56, 58, 59, and 68) and low-risk (6, 11, 40, 42, 43, 44, and 54) types consistent with the generation of squamous cell carcinomas in the uterine cervix^2^. The papillomavirus double-stranded circular DNA genome encodes roughly eight open-reading frames (ORFs), which is responsible for viral replication, maintenance, and cell transformation. These gene products have been classified into six nonstructural regulatory proteins (E1-E6) and two structural capsid proteins (L1 and L2)^3^. Nowadays, the virus-like particles (VLPs) based on L1 proteins have been considered as the best candidate for vaccine development against HPV infections. Two commercial HPV vaccines are both prophylactic. Gardasil which protects against HPV genotypes 6, 11, 16 and 18, and Cervarix which protects against HPV genotypes 16 and 18; Both of them prevent cervical cancer with almost 100% efficacy^4^. These HPV VLP vaccines confer protection against limited genotypes^5^. Although a variable level of cross-protection has also been observed against phylogenetically related HPV genotypes^6^, major impediments such as economic barriers especially gaps in financing obstructed the optimal delivery of vaccines in developing countries. This might be approached via locally produced generic vaccines. Storage and transportation, the so-called cold chain, is another hindrance, which should be mitigated through lyophilization or protectants, and also it should be noted that many recombinant vaccines rely on multiple immunizations; however, promising results could be obtained with a single dose and certainly 2-dose regimens^7^.

Recently, a possible approach to broader cross-type protective immunity at a lower cost is to consider L2-based vaccination compared to L1 VLP immunization^8^. Indeed, the current HPV L2 vaccines improved a type-specific protection. Recent studies showed that some regions in the N-terminus of L2 can neutralize antibodies generated during various types of HPV infections^9^. To overcome the intrinsically low immunogenicity of the recombinant L2 protein, its potency could be increased by various formulations such as the multivalent L2 epitopes (peptide vaccine)^10–12^, fusion with L1 and other immunogenic proteins^13–15^ and multiepitope DNA-based vaccines^16,17^.

As a major field of science, bioinformatics has brought together the concepts of *in silico* analyses of biological queries, mathematics and statistics^18^. Immunoinformatics tools could help researchers to screen multiple HPV genome and predict high immunogenic epitopes, which provide a T or B cell response against HPV infection^19–21^. In this study, the combination of *in silico*/*in vivo* approaches was used to evaluate L1 and L2 proteins of high-risk HPV types (16, 18, 31, 33, 35, 39, 45, 51, 52, 56, 58, 59, and 68), and to design a pan genotype L1 and L2 constructs for development of DNA-based vaccines.

## Results

### Protein conservancy analysis

To select conserved epitopes between HPV subtypes, L1 and L2 protein sequences were aligned using muscle algorithms. Based on the degree of the conservancy, five regions of L1 proteins (8–22, 95–132, 307–342, 398–425 and 449–473) and four regions of L2 proteins (11–40, 54–76, 96–120, 278–305) were selected for further immune-bioinformatics analysis such as B- and T-cell epitope prediction. Among them, region 449–473 of HPV-16 L1 protein and region 54–76 of HPV-16 L2 protein had the highest score of conservancy between all high-risk HPV types. In addition, based on sequence variability of conserved regions, the L1 and L2 proteins from two main types of HPV (16 and 18) were selected as a reference for calculation of conservancy by IEDB epitope conservancy analysis tool (Tables 1 and 2).

**Table 1:** Conservancy analysis of high-risk HPV L1 protein.

Protein Regions	Sequence and degree of conservancy												
8–22 (Type 16)	EATVYLPPVPVSKVV												
	16	18	31	33	35	39	45	51	52	56	58	59	68
	100.00%	60.00%	100.0%	100.0%	93.33%	60.00%	60.00%	60.00%	100.0%	80.00%	100.0%	60.00%	60.00%
8–22 (Type 18)	DNTVYLPPPSVARVV												
	16	18	31	33	35	39	45	51	52	56	58	59	68
	80.00%	100.0%	76.67%	76.67%	76.67%	90.00%	86.67%	83.33%	83.33%	73.33%	83.33%	83.33%	86.67%
95–132 (Type 16)	TQRLVWACVGVEVGRGQPLGVGISGHPLLNKLDDTENA												
	16	18	31	33	35	39	45	51	52	56	58	59	68
	100.00%	81.58%	92.11%	86.84%	92.11%	89.47%	81.58%	84.21%	84.21%	76.32%	81.58%	86.84%	81.58%
95–132 (Type 18)	TQRLVWACAGVEIGRGQPLGVGLSGHPFYNKLDDTESS												
	16	18	31	33	35	39	45	51	52	56	58	59	68
	81.58%	100.0%	78.95%	78.95%	81.58%	81.58%	89.47%	78.95%	81.58%	76.32%	81.58%	89.47%	89.47%
307–342 (Type 16)	FNKPYWLQRAQGHNNGICWGNQLFVTVVDTTRSTNM												
	16	18	31	33	35	39	45	51	52	56	58	59	68
	100.00%	86.11%	97.22%	97.22%	97.22%	86.11%	88.89%	86.11%	100.0%	100.0%	100.0%	83.11%	86.11%
307–342 (Type 18)	FNKPYWLHKAQGHNNGVCWHNQLFVTVVDTTRSTNL												
	16	18	31	33	35	39	45	51	52	56	58	59	68
	86.11%	100.0%	83.33%	83.33%	86.11%	91.67%	97.22%	86.11%	86.11%	86.11%	86.11%	91.67%	91.67%
398–430 (Type 16)	ILEDWNFGLQPPPGGTLEDTYRFVTSQAIACQK												
	16	18	31	33	35	39	45	51	52	56	58	59	68
	100.00%	71.43%	85.71%	78.57%	89.29%	53.57%	67.86%	64.29%	78.57%	60.71%	78.57%	71.43%	60.71%
399–431 (Type 18)	ILEDWNFGVPPPPTTSLVDTYRFVQSVAITCQK												
	16	18	31	33	35	39	45	51	52	56	58	59	68
	71.43%	100.0%	71.43%	71.43%	67.86%	67.86%	96.43%	60.71%	71.43%	60.71%	71.43%	89.29%	75.00%
449–473 (Type 16)	VNLKEKFSADLDQFPLGRKFLLQAG												
	16	18	31	33	35	39	45	51	52	56	58	59	68
	100.00%	84.00%	100.0%	96.00%	96.00%	80.00%	84.00%	80.00%	96.00%	76.00%	96.00%	88.00%	88.00%
450–474 (Type 18)	VDLKEKFSLDLDQYPLGRKFLVQAG												
	16	18	31	33	35	39	45	51	52	56	58	59	68
	84.00%	100.0%	84.00%	88.00%	88.00%	80.00%	96.43%	80.00%	88.00%	88.00%	80.00%	80.00%	84.00%

**Table 2:** Conservancy analysis of high-risk HPV L2 protein.

Protein Regions	Sequence and degree of conservancy												
11–41 (Type 16)	KRASATQLYKTCKQAGTCPPDIIPKVEGKTI												
	16	18	31	33	35	39	45	51	52	56	58	59	68
	100.00%	80.00%	76.67%	83.33%	86.67%	76.67%	80.00%	76.67%	83.33%	73.33%	73.33%	73.33%	73.33%
10–40 (Type 18)	KRASVTDLYKTCKQSGTCPPDVVPKVEGTTL												
	16	18	31	33	35	39	45	51	52	56	58	59	68
	80.00%	100.0%	76.67%	76.67%	76.67%	90.00%	86.67%	83.33%	83.33%	73.33%	83.33%	83.33%	86.67%
96–120 (Type 16)	DPVGPSDPSIVSLVEETSFIDAGAP												
	16	18	31	33	35	39	45	51	52	56	58	59	68
	100.00%	60.0%	76.00%	80.00%	76.00%	64.00%	64.00%	48.00%	64.00%	64.00%	80.00%	68.00%	60.00%
94–118 (Type 18)	EPVGPTDPSIVTLIEDSSVVTSGAP												
	16	18	31	33	35	39	45	51	52	56	58	59	68
	60.00%	100.0%	64.0%	56.00%	52.0%	76.00%	92.00%	52.00%	56.00%	76.00%	60.00%	92.00%	80.00%
54–76 (Type 16)	FFGGLGIGTGSGTGGRTGYIPLG												
	16	18	31	33	35	39	45	51	52	56	58	59	68
	100.00%	95.65%	86.96%	86.96%	86.67%	91.30%	86.96%	91.30%	78.26%	78.26%	95.65%	95.65%	91.30%
53–75 (Type 18)	FLGGLGIGTGSGTGGRTGYIPLG												
	16	18	31	33	35	39	45	51	52	56	58	59	68
	95.65%	100.0%	82.61%	82.61%	82.61%	95.65%	91.30%	95.65%	73.91%	73.91%	91.30%	100.0%	67.86%
278–305 (Type 16)	APDPDFLDIVALHRPALTSRRTGIRYSR												
	16	18	31	33	35	39	45	51	52	56	58	59	68
	100.00%	67.86%	85.71%	78.57%	82.14%	78.57%	64.29%	78.57%	82.14%	75.00%	89.29%	75.00%	82.14%
271–298 (Type 18)	VPDSDFMDIIRLHRPALTSRRGTVRFSR												
	16	18	31	33	35	39	45	51	52	56	58	59	68
	67.86%	100.0%	78.57%	78.57%	78.57%	82.14%	96.43%	85.71%	85.71%	71.43%	78.57%	89.29%	85.71%

### Prediction of linear B-cell epitopes

B-cell epitopes are recognized by B-cell receptors or antibodies in their native structure. Continuous B-cell epitope prediction is very similar to T-cell epitope prediction, which has mainly been based on the amino acid properties such as hydrophobicity, exposed surface area, charge and secondary structure. At first step, the conserved region sequences were analyzed by BepiPred-2 server to predict potential B-cell epitopes (Table 3). In L1 protein, *L1*^8–22^ (EATVYLPPVPVSKVV-type16), L1^408–421^ (PPPGGTLEDTYRFV-type16) and L1^404–417^ (NFGVPPPPTTSLVD-type 18) epitopes had the best B cell epitope identification scores. For L2 protein, L2^22–35^ (KQSGTCPPDVVPKV-type18), L2^100–113^ (PSDPSIVSLVEETS-type16), L2^94–107^ (EPVGPTDPSIVTLI-type18) and L2^57–70^ (GLGIGTGSGTGGRT-type16) epitopes showed the highest epitope identification score between their own protein sequences.

**Table 3:** B-cell epitope identification of HPV L1 & L2 conserved regions.

Protein Name	Regions	B Cell Epitope	Score*
L1	8–22 (Type 16)	EATVYLPPVPVSKVV	1.000
	8–22(Type 18)	DNTVYLPPPSVARV	0.961
	119–132 (Type 16)	GHPLLNKLDDTENA	0.998
	119–132 (Type 18)	GHPFYNKLDDTESS	0.999
	307–320 (Type 16)	FNKPYWLQRAQGHN	0.999
	315–328 (Type 18)	KAQGHNNGVCWHNQ	0.976
	408–421 (Type 16)	PPPGGTLEDTYRFV	1.000
	404–417 (Type 18)	NFGVPPPPTTSLVD	1.000
	454–467 (Type 16)	KFSADLDQFPLGRK	0.999
	547–570 (Type 18)	SLDLDQYPLGRKFL	0.976
L2	25–38 (Type 16)	AGTCPPDIIPKVEG	0.927
	22–35 (Type 18)	KQSGTCPPDVVPKV	1.000
	100–113 (Type 16)	PSDPSIVSLVEETS	1.000
	94–107 (Type 18)	EPVGPTDPSIVTLI	1.000
	57–70 (Type 16)	GLGIGTGSGTGGRT	1.000
	56–69 (Type 18)	GLGIGTGSGTGGRT	1.000
	289–302 (Type 16)	LHRPALTSRRTGIR	0.800
	282–295 (Type 18)	LHRPALTSRRGTVR	0.870

*Higher rates show better quality of epitope identification.

### Prediction of T-cell epitopes

Since a linear form of T-cell epitopes are bound to MHCs, the interface between T-cells and ligands can be accurately modeled. In this study, we used three different algorithms (published motifs, ANN and quantitative matrix) for MHC-I and two algorithms for MHC-II (ANN and quantitative matrix).

### Prediction of MHC-I

At first step, the L1 and L2 conserved regions were analyzed to find the most immunodominant peptides using NetMHCpan 4.0, syfpeithi and ProPred I. In each protein, peptides with the highest binding affinity scores were determined as high-potential CTL epitope candidates (Tables 4 and 5). The analysis showed that L1^12–21^ (YLPPVPVSKV-type 16 and YLPPPSVARV-type 18), L1^460–470^ (DQFPLGRKFLL-type 16), L1^461–471^(DQYPLGRKFLV-type 18), L2^11–20^ (KRASATQLYK-type 16 and KRASVTDLYK-type 18), L2^280–291^ (DPDFLDIVALHR-type 16) and L2^273–284^ (DSDFMDIIRLHR-type 18) epitopes had the highest binding affinity among their own protein sequences. In general, the results of three different algorithms confirmed each other. Conservancy and allergenicity analyses were done on the selected epitopes. The sequence of all the epitopes were well conserved among high-risk HPV types and none of them were allergens (Tables 4 and 5). In addition, there was no cross-reactivity between peptide and human proteome.

**Table 4:** The selected CTL epitopes of HPV L1 protein based on binding affinity.

Protein Name	Position	Epitope Sequence	No. of Alleles	Top alleles	NetMHCpan Average Rank Scores*	ProPred-I Average Scores**	Syfpeithi Average Scores**	Conservancy (=>75%)	Allergenicity
L1	12–21 (Type 16)	YLPPVPVSKV	17	HLA-A02:01 HLA-A03:01 HLA-B07:02	0.574	97.324	17.285	Type 31: 100% Type 33: 100% Type 35: 100% Type 51: 100% Type 52: 100% Type 58: 100% Type 56: 90%	Non-allergen
	12–21 (Type 18)	YLPPPSVARV	17	HLA-A02:01 HLA-A03:01 HLA-A26:01	0.616	96.521	16.818	Type 45: 100% Type 39: 90% Type 59: 90% Type 68: 90%	Non-allergen
	114–125 (Type 16)	GVGISGHPLLNK	13	HLA-A03:01 HLA-B58:01	0.911	61.956	13.673	Type 31: 100% Type 33: 100% Type 35: 100% Type 52: 100% Type 51: 83.33% Type 58: 83.33%	Non-allergen
	104–115 (Type 18)	GVEIGRGQPLGV	12	HLA-B08:01 HLA-B39:01 HLA-B40:01	0.831	64.810	13.678	Type 59: 100% Type 68: 100% Type 39: 91.67% Type 45: 91.67% Type 51: 91.67% Type 58: 91.67% Type 56: 75%	Non-allergen
	114–125 (Type 18)	GVGLSGHPFYNK	11	HLA-A01:01 HLA-A03:01 HLA-B58:01	0.840	63.982	14.129	Type 45: 91.67% Type 59: 91.67% Type 51: 83.33% Type 68: 83.33%	Non-allergen
	411–421 (Type 16)	GGTLEDTYRFV	14	HLA-A01:01 HLA-A02:01 HLA-A03:01	0.789	68.232	14.750	Type 31: 81.82% Type 33: 63.64% Type 35: 81.82%	Non-allergen
	414–425 (Type 18)	PTTSLVDTYRFV	14	HLA-A01:01 HLA-A02:01 HLA-A03:01	0.850	75.541	15.868	Type 45: 100% Type 59: 91.67% Type 33: 75% Type 52: 75% Type 58: 75%	Non-allergen
	460–470 (Type 16)	DQFPLGRKFLL	16	HLA-B08:01 HLA-B07:02 HLA-A24:02 HLA-A26:01 HLA-B39:01	0.757	79.497	16.523	Type 31: 100% Type 33: 100% Type 35: 100% Type 39: 100% Type 52: 100% Type 58: 100% Type 59: 100% Type 68: 100% Type 51: 90.91% Type 56: 90.91% Type 18: 81.82% Type 45: 81.82%	Non-allergen
	461–471 (Type 18)	DQYPLGRKFLV	17	HLA-B08:01 HLA-B07:02 HLA-A24:02 HLA-A26:01 HLA-B39:01	0.723	129.350	16.531	Type 45: 100% Type 16: 81.82% Type 31: 81.82% Type 33: 81.82% Type 35: 81.82% Type 39: 81.82% Type 52: 81.82% Type 56: 81.82% Type 58: 81.82% Type 59: 81.82% Type 68: 81.82%	Non-allergen

*lower rates show better binding affinity, **Higher rates show better binding affinity.

**Table 5:** The selected CTL epitopes of HPV L2 Protein, based on binding affinity.

Protein Name	Position	Epitope Sequence	No. of Alleles	Top alleles	NetMHCpan Average Rank Scores*	ProPred-I Average Scores**	Syfpeithi Average Scores**	Conservancy (= > 75%)	Allergenicity
L2	11–20 (Type 16)	KRASATQLYK	17	HLA-A03:01 HLA-B58:01 HLA-B15:01 HLA-B27:05	0.576	413.070	17.280	Type 31: 100% Type 33: 100% Type 35: 100% Type 51: 100% Type 52: 100% Type 58: 100% Type 56: 90%	Non-allergen
	11–20 (Type 18)	KRASVTDLYK	17	HLA-A03:01 HLA-B58:01 HLA-B15:01 HLA-B27:05	0.626	415.190	17.360	Type 31: 100% Type 33: 100% Type 35: 100% Type 51: 100% Type 52: 100% Type 58: 100% Type 56: 90%	Non-allergen
	60–71 (Type 18)	GTGSGTGGRTGY	13	HLA-A03:01 HLA-A01:01 HLA-B15:01	1.502	33.7167	11.585	Type 31: 100% Type 33: 100% Type 35: 100% Type 51: 100% Type 52: 100% Type 58: 100% Type 56: 90%	Non-allergen
	280–291 (Type 16)	DPDFLDIVALHR	12	HLA-A01:01 HLA-A03:01 HLA-A26:01	1.198	53.281	15.375	Type 31: 100% Type 33: 100% Type 35: 100% Type 51: 100% Type 52: 100% Type 58: 100% Type 56: 90%	Non-allergen
	293–303 (Type 16)	ALTSRRTGIRY	10	HLA-A01:01 HLA-A15:01 HLA-A26:01	0.873	42.308	14.720	Type 31: 100% Type 33: 100% Type 35: 100% Type 51: 100% Type 52: 100% Type 58: 100% Type 56: 90%	Non-allergen
	273–284 (Type 18)	DSDFMDIIRLHR	12	HLA-A40:01 HLA-A01:01 HLA-A03:01	1.031	65.570	14.754	Type 31: 100% Type 33: 100% Type 35: 100% Type 51: 100% Type 52: 100% Type 58: 100% Type 56: 90%	Non-allergen
	286–296 (Type 18)	ALTSRRGTVRF	10	HLA-A58:01 HLA-B27:05 HLA-B08:01	0.845	43.607	14.590	Type 31: 100% Type 33: 100% Type 35: 100% Type 51: 100% Type 52: 100% Type 58: 100% Type 56: 90%	Non-allergen

*lower rates show better binding affinity, **Higher rates show better binding affinity.

### Prediction of MHC-II

In this study, we used NetMHCIIpan and Propred servers for MHC-II epitope identification analysis (Table 6). Since a suitable T-cell epitope should be predicted to bind to different HLA alleles, epitopes with the maximum number of binding HLA-DR alleles were selected as high-potential helper T-cell epitope candidates. Among predicted epitopes, L1^8–22^ (EATVYLPPVPVSKVV-type 16), L1^95–111^ (TQRLVWACVGVEVGRGQ-type 16 and TQRLVWACAGVEIGRGQ-type 18), L1^416–430^ (DTYRFVTSQAIACQK-type 16), L1^417–431^ (DTYRFVQSVAITCQK-type 18), L2^100–118^ (DPSIVTLIEDSSVVTSGAP-type 16), L2^281–297^ (PDFLDIVALHRPALTSR-type 16) and L2^274–290^ (SDFMDIIRLHRPALTSR-type 18) had the highest scores of binding affinity. Also, the sequence of all the epitopes were well conserved among high-risk HPV types and none of them were allergen (Tables 6 and 7). Also, there was no cross-reactivity between peptide and human proteome.

**Table 6:** The selected helper T Cell epitopes of HPV L1 protein based on binding affinity.

Protein Name	Position	Epitope Sequence	No. of Alleles	Top Alleles	NetMHCIIpan Average Rank Scores*	ProPred II Average Scores**	Conservancy (= > 75%)	Allergenicity
L1	8–22 Type 16	EATVYLPPVPVSKVV	13	DQA:10103 DRB1:1001 DQA:10201 DRB1:0101	6.666	2.735	Type 31: 100% Type 33: 100% Type 52: 100% Type 58: 100% Type 35: 93.3% Type 56: 80%	Non-allergen
	8–22 Type 18	DNTVYLPPPSVARVV	12	DQA:10501 DRB1:0101 DRB1:1602	6.250	3.180	Type 45: 93.3% Type 59: 86.6% Type 68: 86.6% Type 39: 80%	Non-allergen
	95–111 Type 16	TQRLVWACVGVEVGRGQ	10	DRB1:0401 DRB1:0403 DQA:10301	6.630	2.790	Type 39: 100% Type 31: 94.1% Type 33: 88.2% Type 35: 88.2% Type 58: 88.2% Type 59: 88.2% Type 68: 88.2% Type 51: 88.2% Type 18: 88.2% Type 45: 88.2% Type 52: 82.3% Type 56: 82.3%	Non-allergen
	95–111 Type 18	TQRLVWACAGVEIGRGQ	9	DRB1:0402 DRB1:0403 DQA:10301	6.633	2.720	Type 39: 88.2% Type 31: 88.2% Type 33: 88.2% Type 58: 88.2% Type 59: 88.2% Type 68: 88.2% Type 16: 88.2% Type 45: 88.2% Type 52: 88.2%	Non-allergen
	327–342 Type 16	NQLFVTVVDTTRSTNM	14	DRB1:0401 DRB1:0405 DRB1:0802 DQA:10201	6.940	2.110	Type 31: 100% Type 35: 100% Type 52: 100% Type 56: 100% Type 58: 100% Type 18: 93.7% Type 33: 93.7% Type 45: 93.7% Type 68: 88.2% Type 39: 87.5% Type 59: 87.5%	Non-allergen
	324–342 Type 18	NQLFVTVVDTTRSTNL	14	DRB1:0401 DRB1:0405 DRB1:0701 DRB1:0802	6.690	2.127	Type 45: 100% Type 16: 93.7% Type 31: 93.7% Type 35: 93.7% Type 52: 93.7% Type 56: 93.7% Type 58: 93.7% Type 59: 93.7% Type 33: 87.5% Type 39: 87.5% Type 51: 87.5%	Non-allergen
	416–430 Type 16	DTYRFVTSQAIACQK	34	DRB1:0701 DRB1:0101 DRB1:0802 DRB1:0401	3.371	5.521	Type 31: 93.3% Type 33: 93.3% Type 58: 93.3% Type 52: 86.6% Type 18: 80% Type 35: 80% Type 51: 76.6%	Non-allergen
	417–431 Type 18	DTYRFVQSVAITCQK	34	DRB1:0701 DRB1:0101 DRB1:0802 DRB1:0401	3.385	5.364	Type 45: 93.3% Type 31: 86.6% Type 33: 86.6% Type 52: 86.6% Type 58: 86.6% Type 59: 86.6% Type 68: 80% Type 16: 80% Type 56: 76.6%	Non-allergen

*lower rates show better binding affinity, **Higher rates show better binding affinity.

**Table 7:** The selected helper T Cell epitopes of HPV L2 protein, based on binding affinity.

Protein Name	Position	Epitope Sequence	No. of Alleles	Top Alleles	NetMHCIIpan Average Rank Scores*	ProPred II Average Scores**	Conservancy (= > 75%)	Allergenicity
L2	102–120 Type 16	DPSIVSLVEETSFIDAGAP	9	DQA:10101 DQA:10102	8.120	1.257	Type 33: 84.2% Type 35: 84.2% Type 58: 84.2%	Non-allergen
	100–118 Type 18	DPSIVTLIEDSSVVTSGAP	12	DRB1:0401 DRB1:0301 DRB3:0101	7.980	1.315	Type 45: 89.4% Type 59: 89.4% Type 39: 75.6% Type 51: 75.6%	Non-allergen
	54–69 Type 16	FFGGLGIGTGSGTGGR	8	DRB1:0402 DQA:10301 DQA:10501	7.160	1.645	Type 58: 100% Type 18: 93.7% Type 31: 93.7% Type 33: 93.7% Type 35: 93.7% Type 59: 93.7% Type 68: 93.7% Type 39: 87.5% Type 45: 87.5% Type 51: 87.5% Type 52: 87.5% Type 56: 81.2%	Non-allergen
	281–297 Type 16	PDFLDIVALHRPALTSR	32	DRB5:0101 DRB4:0101 DRB1:0801 DRB1:0402	4.165	2.148	Type 58: 100% Type 31: 94.1% Type 39: 94.1% Type 52: 94.1% Type 68: 94.1% Type 33: 88.2% Type 35: 88.2% Type 51: 88.2% Type 59: 88.2% Type 16: 76.4% Type 56: 76.4%	Non-allergen
	274–290 Type 18	SDFMDIIRLHRPALTSR	26	DRB1:0101 DRB1:0103 DRB1:0801 DRB1:1501	3.493	2.864	Type 45: 94.1% Type 35: 88.2% Type 59: 88.2% Type 31: 82.3% Type 39: 82.3% Type 51: 82.3% Type 52: 82.3% Type 68: 82.3% Type 16: 76.4% Type 33: 76.4% Type 58: 76.4%	Non-allergen

*lower rates show better binding affinity, **Higher rates show better binding affinity.

### Tap transport/proteasomal cleavage

The generation of antigenic peptides and their transport across the membrane of the endoplasmic reticulum for assembly with MHC class I molecules are essential steps in antigen presentation to cytotoxic T lymphocytes. Thus, investigating the proteasomal cleavage, Tap transport and affinity prediction of binding is essential in MHC-1 presentation pathway. The NetCTL2.1 server was used to predict TAP transport efficiency and proteasomal cleavage scores (Table 8). Between all epitopes, L1^12–21^ (YLPPVPVSKV-type 16 and YLPPPSVARV-type 18), L1^460–470^ (DQFPLGRKFLL-type 16), L1^461–471^ (DQYPLGRKFLV-type 18), L2^11–20^ (KRASATQLYK-type 16 and KRASVTDLYK-type 18), L2^293–303^ (DPDFLDIVALHR-type 16) and L2^273–284^ (DSDFMDIIRLHR-type 18) epitopes had the highest epitope identification scores.

**Table 8:** Proteasomal cleavage and TAP transport efficiency scores of MHC-I predicted epitopes.

Protein Name	Position	Epitope Sequence	Proteasomal C terminal cleavage Score*	TAP transport efficiency Score**	Epitope identification Score***
L1	12–21 (Type 16)	YLPPVPVSKV	1.0473	0.9771	1.3162
	12–21 (Type 18)	YLPPPSVARV	0.9753	0.8581	0.9656
	114–125 (Type 16)	GVGISGHPLLNK	0.8023	0.6695	0.7094
	104–115 (Type 18)	GVEIGRGQPLGV	0.5253	0.9200	0.7017
	114–125 (Type 18)	GVGLSGHPFYNK	0.3726	0.9360	0.6484
	411–421 (Type 16)	GGTLEDTYRFV	0.5795	0.9599	0.8396
	414–425 (Type 18)	PTTSLVDTYRFV	0.5992	0.9267	0.7579
	460–470 (Type 16)	DQFPLGRKFLL	0.9879	0.9810	1.2046
	461–471 (Type 18)	DQYPLGRKFLV	0.9358	0.9606	1.0779
L2	11–20 (Type 16)	KRASATQLYK	1.3081	0.9773	1.6173
	11–20 (Type 18)	KRASVTDLYK	1.1145	0.9726	1.4230
	60–71 (Type 18)	GTGSGTGGRTGY	0.4471	0.4000	0.5630
	280–291 (Type 16)	DPDFLDIVALHR	0.8251	0.9405	1.0209
	293–303 (Type 16)	ALTSRRTGIRY	0.6667	0.9280	0.8580
	273–284 (Type 18)	DSDFMDIIRLHR	0.6514	0.9917	0.8881
	286–296 (Type 18)	ALTSRRGTVRF	0.6545	0.8565	0.7305

*Higher rates show better quality of proteasomal cleavage, **Higher rates show better quality of tap transport efficiency, *** Higher rates show better quality of epitope identification.

### Population coverage analysis

HLA distribution varies among the diverse geographic regions around the world. Thus, while designing an effective vaccine, population coverage must be taken into consideration to cover the maximum possible populations. In this study, population coverage was estimated separately for each putative epitope in 16 specified geographic regions of the world (Tables 9 and 10). For CTL epitopes, the highest population coverage of world’s population was calculated for L1^12–21^ (84.71%), L1^411–421^ (90.87%), L2^11–20^ (73.89%) and L2^280–291^ (67.72%). For helper T-cell epitopes, the highest population coverage was calculated 86.18% for L1^8–22^, 91.18% for L1^327–342^, 98.90% for L1^416–430^, 83.47% for L2^100–118^ and 97.68% for L2^281–297^. Overall, these results indicated that high-potential helper T-cell epitopes and CTL epitopes can specifically bind to the prevalent HLA molecules in the target populations where the vaccine will be employed.

**Table 9:** Population coverage of putative HPV L1 and L2 CTL epitopes.

Area	L1^12–21^ (16)	L1^12–21^ (18)	L1^114–125^ (16)	L1^104–115^ (18)	L1^114–125^ (18)	L1^411–421^ (16)	L1^414–425^ (18)	L1^460–470^ (16)	L1^461–471^ (18)	L2^11–20^ (16)	L2^11–20^ (18)	L2^60–71^ (18)	L2^280–291^ (16)	L2^273–284^ (18)	L2^293–303^ (16)	L2^286–296^ (18)
Central Africa	55.19%	55.19%	34.15%	22.23%	35.05%	40.96%	49.52%	34.22%	36.11%	42.77%	34.54%	24.31%	35.98%	37.81%	33.67%	34.62%
East Africa	54.55%	54.55%	39.30%	27.20%	36.15%	51.59%	54.39%	30.43%	32.11%	49.58%	42.87%	25.16%	36.61%	42.47%	36.72%	39.59%
East Asia	84.07%	84.07%	30.67%	26.13%	46.90%	86.10%	85.50%	81.17%	84.54%	54.35%	40.97%	36.22%	43.43%	42.51%	54.30%	46.27%
Europe	89.34%	89.34%	60.82%	55.22%	37.79%	88.70%	96.10%	76.80%	89.95%	80.93%	68.96%	63.88%	78.16%	77.28%	80.28%	64.53%
North Africa	68.64%	68.64%	37.64%	32.15%	69.17%	60.63%	69.98%	39.29%	57.62%	55.29%	46.79%	32.60%	46.71%	45.61%	45.63%	45.67%
North America	85.51%	85.51%	48.90%	43.94%	50.77%	83.68%	89.30%	65.12%	82.23%	73.49%	53.90%	44.74%	61.85%	61.35%	61.91%	62.58%
Northeast Asia	79.00%	79.00%	38.67%	23.45%	53.09%	70.67%	81.43%	76.55%	62.69%	69.49%	63.65%	57.63%	65.89%	66.78%	49.11%	63.87%
Oceania	84.82%	84.82%	50.06%	31.64%	60.43%	83.11%	92.01%	85.37%	78.07%	53.40%	51.71%	48.49%	71.38%	70.41%	56.81%	50.88%
South America	78.25%	78.25%	20.45%	25.76%	52.77%	63.18%	68.59%	43.70%	56.26%	46.21%	33.69%	30.33%	38.41%	37.14%	37.62%	33.33%
South Asia	72.15%	72.15%	34.84%	26.57%	25.66%	62.49%	77.44%	59.68%	51.83%	63.27%	65.43%	56.50%	61.15%	63.04%	49.59%	59.77%
Southeast Asia	78.86%	78.86%	53.56%	34.96%	61.28%	80.88%	81.80%	77.47%	72.71%	60.06%	51.86%	42.69%	65.29%	67.68%	51.02%	54.58%
Southwest Asia	70.47%	70.47%	31.98%	25.26%	52.05%	66.54%	78.02%	52.27%	63.36%	59.00%	50.51%	43.25%	53.69%	54.06%	49.32%	44.15%
West Africa	62.72%	62.72%	36.52%	30.77%	55.77%	52.15%	60.43%	36.13%	46.89%	50.41%	41.76%	28.75%	42.12%	44.16%	42.38%	45.54%
West Indies	77.79%	77.79%	47.01%	37.22%	53.81%	76.69%	85.20%	61.18%	75.51%	61.85%	49.30%	43.11%	62.31%	61.66%	62.60%	50.22%
World	**84**.**71%**	**84**.**71%**	**49**.**45%**	**41**.**88%**	**59**.**28%**	**82**.**59%**	**90**.**87%**	**69**.**64%**	**81**.**88%**	**73**.**89%**	**60**.**46%**	**54**.**30%**	**67**.**72%**	**67**.**23%**	**66**.**33%**	**57**.**18%**

**Table 10:** Population coverage of putative HPV L1 and L2 helper T-cell epitopes.

Area	L1^8–22^ (16)	L1^8–22^ (18)	L1^95–111^ (16)	L1^95–111^ (18)	L1^327–342^ (16)	L1^324–342^ (18)	L1^416–430^ (16)	L1^417–431^ (18)	L2^102–120^ (16)	L2^100–118^ (18)	L2^59–49^ (16)	L2^281–297^(16)	L2^274–290^ (18)
Central Africa	65.08%	65.08%	64.35%	64.35%	90.94%	75.69%	99.39%	99.39%	80.48%	83.43%	49.31%	98.60%	89.90%
East Africa	66.54%	66.54%	70.11%	70.11%	90.05%	77.27%	99.99%	99.99%	88.08%	90.13%	53.65%	98.69%	90.60%
East Asia	69.98%	69.98%	75.84%	75.84%	69.37%	66.07%	88.71%	88.71%	59.49%	65.82%	62.40%	76.50%	83.92%
Europe	88.93%	88.93%	79.32%	79.32%	99.02%	75.55%	99.89%	99.89%	78.99%	87.12%	67.21%	99.73%	91.82%
North Africa	84.09%	84.09%	83.15%	81.15%	82.47%	82.47%	98.06%	98.06%	81.95%	87.81%	75.06%	87.75%	87.34%
North America	92.98%	92.98%	95.53%	95.53%	99.72%	62.42%	99.98%	99.98%	80.87%	88.15%	90.22%	99.97%	93.09%
Northeast Asia	80.21%	80.21%	77.53%	78.53%	90.36%	69.48%	97.98%	97.98%	79.79%	81.80%	79.68%	92.97%	76.27%
Oceania	78.65%	78.65%	81.02%	81.02%	87.29%	59.42%	98.48%	98.48%	82.52%	82.94%	73.46%	95.17%	68.62%
South America	76.77%	76.77%	96.22%	95.22%	83.61%	47.14%	98.49%	98.49%	68.33%	72.91%	89.31%	97.33%	70.54%
South Asia	92.78%	92.78%	74.94%	74.94%	97.54%	85.17%	99.78%	99.78%	85.81%	89.61%	73.29%	97.39%	87.64%
Southeast Asia	72.82%	72.82%	61.75%	60.75%	61.99%	61.99%	91.77%	91.77%	66.82%	69.91%	55.15%	73.24%	71.50%
Southwest Asia	76.19%	76.19%	70.78%	70.78%	64.31%	64.31%	98.54%	98.54%	78.54%	82.11%	71.73%	73.11%	64.28%
West Africa	81.06%	81.06%	85.85%	85.85%	78.49%	65.12%	99.79%	99.79%	91.65%	92.88%	70.26%	97.64%	86.49%
West Indies	84.64%	84.64%	74.91%	74.91%	82.74%	82.74%	99.89%	99.89%	91.03%	92.62%	67.03%	84.73%	85.35%
World	**86**.**18%**	**86**.**18%**	**80**.**46%**	**80**.**36%**	**91**.**18%**	**70**.**83%**	**98**.**90%**	**98**.**90%**	**76**.**16%**	**83**.**74%**	**70**.**79%**	**97**.**68%**	**89**.**75%**

### Peptide-protein flexible Docking

**Figure 1:**
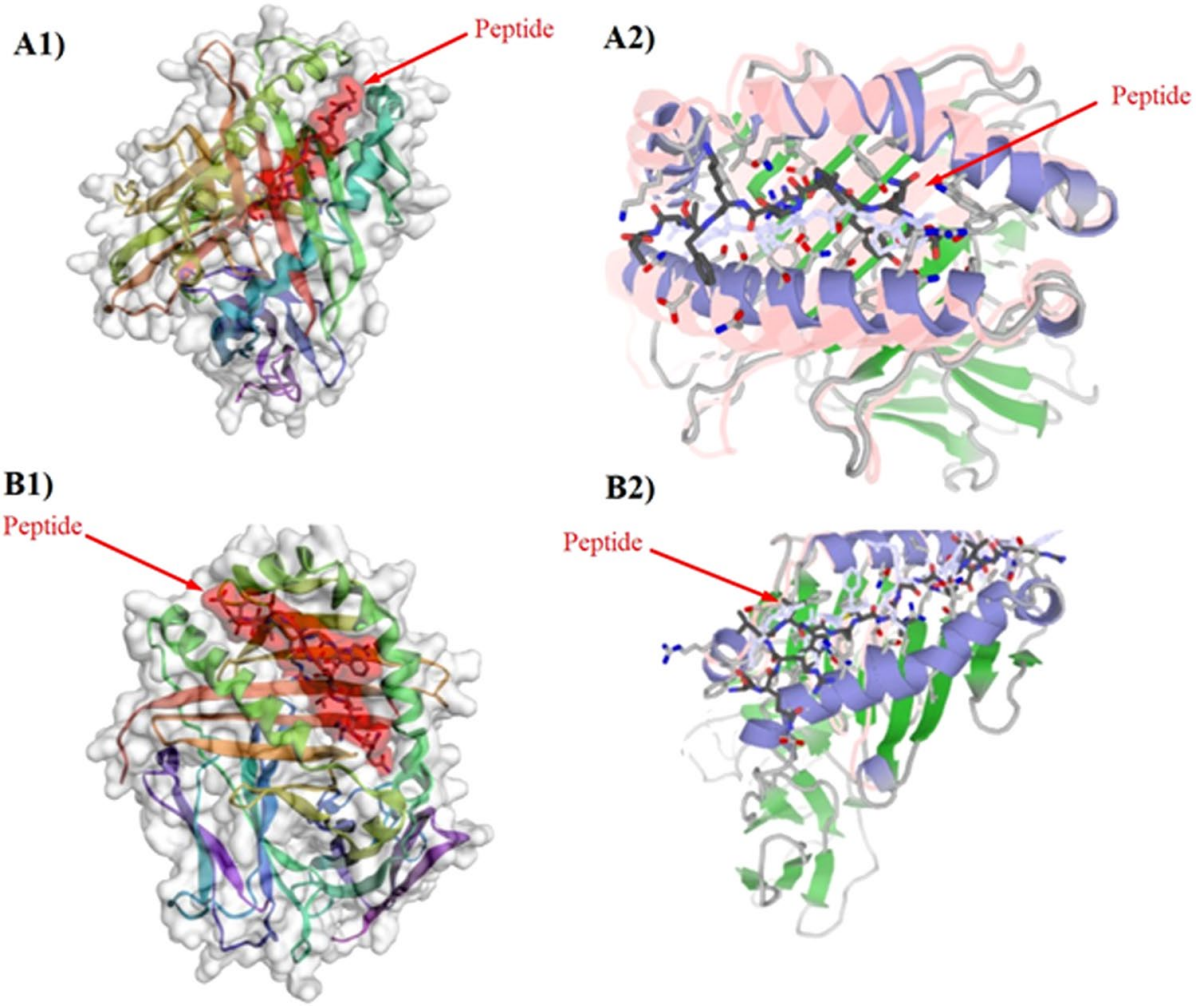
(**A1**) Successful peptide-protein Docking between L1^12–21^ (YLPPVPVSKV) and HLA 0301 with cluster density scores of 214.7; (**A2**) Successful peptide-protein Docking between L1^12–21^ (YLPPVPVSKV) and HLA 0301 with interaction similarity scores of 215; (**B1**) Successful peptide-protein Docking between L2^100–118^ and HLA DRB1–0101 with cluster density of 121.06; (**B2**) Successful peptide-protein Docking between L2^100–118^ and HLA DRB1–0101 with interaction similarity scores of 146.0.

Peptides are promising candidates for different types of biological applications such as vaccine design. In recent years, a variety of approaches have been revealed for ‘protein-peptide docking, which is, predicting the structure of the protein-peptide complex, starting from the protein structure and the peptide sequence, including variable degrees of information about the peptide binding site and/or conformation. In this study, two different algorithms (Template-based and global docking) were used to calculate docking scores between MHC allele and peptides. At first, structure data of MHC-I and MHC-II were downloaded from RCSB PDB server (https://www.rcsb.org/). Then, all potential epitopes and MHC PDB files were submitted to the server separately. Top model with the highest cluster density (number of elements divided by average cluster RMSD, obtained from CABS-dock server) and interaction similarity score (similarity of the amino acids of the target complex aligned to the contacting residues in the template structure to the template amino acids, obtained from GalexyPepDock server) were selected for each peptide and its MHC (Tables 11 and 12). The results in each MHC allele might vary but in average scores, similarity score and cluster density confirmed each other. For CTL epitope, L1^12–21^, L1^104–115^, L1^460–470^, L2^11–20^, L2^280–291^, L2^273–284^ had the highest average docking scores on both servers. For helper T-cell epitope, L1^8–22^, L1^95–111^, L1^417–431^, L2^100–118^, L2^59–49^ and L2^274–290^ had the highest average docking scores on both servers. Figure 1 represents a sample of successful docking model between peptide and MHC protein (successful docking means epitope binding to an MHC molecule through interaction between their R group of side chains and pockets located on the floor of the MHC molecule). Moreover, Table 13 shows MHC allele used for peptide-protein docking.

**Table 11:** MHC-I -peptide docking scores of selected CTL epitopes.

Epitope (Type)	HLA A0101		HLA A0201		HLA A0301		HLA A2402		HLA A1101		HLA B0702		HLA B0801		HLA B2705		HLA B3501		HLA B5101		Average	
	C.D*	I.S*	C.D	I.S	C.D	I.S	C.D	I.S	C.D	I.S	C.D	I.S	C.D	I.S	C.D	I.S	C.D	I.S	C.D	I.S	C.D	I.S
L1^12–21^ (16)	62.70	262	48.87	216	214.7	215	67.20	240	74.46	229	123.04	275	59.94	250	51.52	239	166.52	296	200.92	322	106.79	254.4
L1^12–21^ (18)	63.52	256	44.63	221	90.0	212	80.77	238	40.71	236	73.88	285	58.79	260	200.4	253	142.3	300	72.66	336	86.77	259.7
L1^114–125^ (16)	31.43	208	96.97	229	52.27	174	64.62	185	47.22	198	42.94	198	63.84	206	74.77	210	97.73	199	84.57	194	65.64	200.1
L1^104–115^ (18)	93.03	207	255.2	231	64.10	176	40.71	180	69.53	175	68.26	188	57.74	208	82.24	186	38.35	199	48.11	212	81.73	196.2
L1^114–125^ (18)	70.28	207	52.22	231	81.57	176	67.66	180	70.79	175	105.7	188	74.03	208	111.9	199	71.94	185	61.52	212	76.75	196.1
L1^411–421^ (16)	86.44	210	49.32	216	28.77	162	57.40	184	38.22	177	73.79	180	48.97	201	41.36	167	46.61	191	29.67	197	49.75	188.5
L1^414–425^ (18)	69.15	186	80.12	226	32.33	170	47.60	193	45.81	188	54.46	186	86.69	198	49.10	186	71.61	210	116.21	186	65.01	192.9
L1^460–470^ (16)	34.38	244	319.1	244	91.4	244	71.11	233	62.62	242	41.82	256	96.85	286	64.73	251	53.76	280	56.76	258	94.93	251.8
L1^461–471^ (18)	49.32	215	71.94	263	134.6	211	58.35	212	68.53	214	100.2	229	39.65	238	46.39	224	66.01	253	184.38	232	81.94	229.1
L2^11–20^ (16)	22.54	170	99.89	155	70.24	147	38.76	156	119.5	152	23.31	182	108.6	175	95.11	195	100.0	198	43.29	185	74.14	171.5
L2^11–20^ (18)	30.70	175	101.5	156	73.11	145	33.14	157	64.31	155	43.48	182	44.14	171	68.18	190	121.3	199	56.07	188	63.60	171.8
L2^60–71^ (18)	31.70	176	41.61	167	32.51	161	40.97	172	37.48	179	90.20	203	66.10	199	42.57	190	75.45	220	51.26	202	50.98	186.9
L2^280–291^(16)	79.13	240	42.95	204	11.33	185	30.04	210	148.6	198	72.19	204	66.63	224	275.2	197	20.27	212	82.55	197	82.89	207
L2^273–284^(18)	44.79	235	49.99	199	35.35	186	50.91	206	20.74	201	43.92	200	118.9	217	80.86	195	53.13	195	363.6	184	86.23	201.7
L2^293–303^(16)	27.98	170	52.31	176	62.58	180	43.49	177	56.57	179	103.4	220	62.86	199	48.09	190	69.012	185	50.69	189	57.70	186.5
L2^286–296^(18)	18.79	233	44.04	195	141.7	175	31.84	182	40.80	185	42.40	219	131.2	234	26.61	202	73.97	199	48.86	191	60.03	201.5

*higher rate shows better quality of peptide-MHC interactions.

**Table 12:** MHC-II -peptide docking scores of selected helper T-cell epitopes.

Epitope (Type)	DRB1–0101		DRB1–0301		DRB1–0401		DRB1–1101		DRB1–1501		DRB5–0101		Average	
	C.D*	I.S*	C.D	I.S	C.D	I.S	C.D	I.S	C.D	I.S	C.D	I.S	C.D	I.S
L1^8–22^ (16)	48.841	149	463.013	120	33.772	120	41.410	118	30.990	118	47.773	119	60.966	124
L1^8–22^ (18)	57.344	137	45.1999	125	48.297	125	35.110	125	38.195	125	53.419	125	46.261	127
L1^95–111^ (16)	24.887	136	11.423	136	40.015	136	12.880	136	22.660	136	53.440	136	27.551	136
L1^95–111^ (18)	51.199	136	89.320	136	29.488	136	26.401	136	12.182	136	32.170	136	40.127	136
L1^327–342^ (16)	40.692	123	44.417	117	139.393	115	73.978	115	28.265	115	70.108	110	66.142	115.8
L1^324–342^ (18)	54.704	123	62.131	117	39.789	114	14.761	114	21.197	114	156.779	110	56.227	115.3
L1^416–430^ (16)	47.280	135	166.768	133	55.994	135	12.043	135	13.596	133	40.599	135	56.047	134.3
L1^417–431^ (18)	131.173	147	47.635	147	35.827	147	51.537	147	20.434	147	88.819	147	62.571	147
L2^102–120^ (16)	43.581	118	34.194	120	46.564	133	19.9137	129	112.397	149	28.873	118	47.588	127.8
L2^100–118^ (18)	121.068	146	29.429	130	59.920	128	40.538	128	25.8673	128	41.720	128	53.090	131.3
L2^59–49^ (16)	72.663	116	100.837	110	33.013	116	36.984	116	75.634	116	71.575	166	65.117	115
L2^281–297^(16)	46.236	139	36.971	139	138.283	139	56.161	139	49.871	139	44.997	139	62.086	139
L2^274–290^ (18)	62.014	139	58.308	139	146.188	139	36.501	139	41.267	139	61.209	139	67.581	139

*higher rate shows better quality of peptide-MHC interactions.

**Table 13:** MHC allele used for peptide – protein docking.

MHC-I		MHC-II	
Allele	PDB code	Allele	PDB code
HLA-A01:01	4NQV	DRB1:0101	4AH2
HLA-A02:01	4UQ3	DRB1:0301	2Q6W
HLA-A03:01	3RL2	DRB1:0401	5LAX
HLA-A11:01	1 × 7Q	DRB1:1101	6CPL
HLA-A24:02	5HGA	DRB1:1501	5V4M
HLA-B07:02	5EO1	DRB5:0101	1FV
HLA-B08:01	3SPV		
HLA-B27:05	1OGT		
HLA-B35:01	3LKN		
HLA-B51:01	1E27		

### Construct design

**Figure 2:**
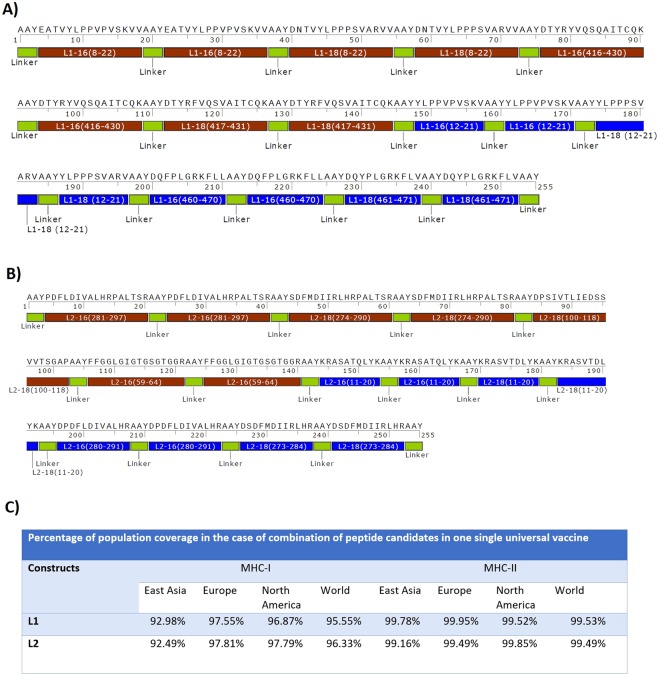
(**A**) L1 construct, (**B**) L2 construct, (**C**) Percentage of population coverage in the combination of peptide candidates in one single universal construct.

After performing the analysis, top-ranked epitopes were selected according to these parameters: (1) binding affinity between peptide and MHC (for both MHC-I and II alleles), (2) epitope identification scores for T- and B-cells, (3) proteasomal cleavage and tap transport scores, (4) conservancy degree between HPV subtypes, (5) population coverage, and (6) scores of peptide-protein docking. Based on L1 and L2 top-ranked epitopes, two different constructs were designed (Fig. 2). For L1 structure, L1^12–21^ (type 16 & 18), L1^460–470^ (type 16), L1^461–471^ (type 18), L1^8–22^ (type 16 & 18), L1^416–430^ (type 16) and L1^417–431^ (type 18) epitopes were selected. For L2 structure, L2^11–20^ (type 16 & 18), L2^280–291^ (type 16), L2^273–284^ (type 18), L2^281–297^ (type 16), L2^274–290^ (type 18) and L2^54–69^ (type 16) epitopes were presented (Table 14). For both structures, two repeats of each epitope were placed together with AAY proteolytic linker (alanine, alanine, and tyrosine). Physicochemical properties of L1 and L2 constructs (molecular weight, instability index, antigenicity, solubility and estimated half time) were summarized in Table 15.

**Table 14:** *In silico* analysis of top ranked epitopes.

Epitope sequence (HPV-Type)	Protein	Average MHC binding affinity scores (top MHC alleles)		Epitope identification scores		Proteasomal cleavage and tap transport scores		Conservancy degree between hrHPV types (Average)	Population coverage (world)	Scores of peptide-protein docking.	
		MHC-I	MHC-II	B Cell	T Cell	Tap Score	Proteasomal score			C.D	I.S
EATVYLPPVPVSKVVA (16)	L1		6.666 (HLA-DQA10103)	1.000				95.55%	86.18%	60.966	124
DNTVYLPPPSVARV (18)	L1		6.250 (HLA-DQA10501)	0.961				86.62%	86.18%	46.261	127
DTYRFVTSQAIACQK (16)	L1		3.371 (DRB1:0701)	0.999				86.15%	98.90%	56.047	134.3
DTYRFVQSVAITCQK (18)	L1		3.385 (DRB1:0701)	1.000				84.76%	98.90%	62.571	147
YLPPVPVSKV (16)	L1	0.574 (HLA-A*02:01)			1.316	1.047	0.977	98.57%	84.71%	106.79	254.4
YLPPPSVARV (18)	L1	0.616 (HLA-A*02:01)			0.965	0.975	0.858	92.50%	84.71%	86.77	259.7
DQFPLGRKFLL (16)	L1	0.757 (HLA-B*08:01)			1.204	0.987	0.981	95.45%	69.64%	94.93	251.8
DQYPLGRKFLV (18)	L1	0.723 (HLA-B*08:01)			1.077	0.936	0.960	83.47%	81.88%	81.94	229.1
PDFLDIVALHRPALTSR (16)	L2		4.165 (DRB5:0101)	0.870				89.27%	83.74%	62.086	139
SDFMDIIRLHRPALTSR (18)	L2		3.493 (DRB1:0103)	1.000				82.83%	70.79%	67.581	139
FFGGLGIGTGSGTGGR (16)	L2		7.160 (DRB1:0402)	1.000				82.50%	97.68%	65.117	115
DPSIVTLIEDSSVVTSGAP (18)	L2		7.980 (DRB3:0101)	1.000				91.11%	89.75%	53.090	131.3
KRASATQLYK (16)	L2	0.576 (HLA-A*03:01)			1.617	1.308	0.977	98.57%	73.89%	74.14	171.5
KRASVTDLYK (18)	L2	0.626 (HLA-B*27:05)			1.423	1.114	0.972	98.57%	60.46%	63.60	171.8
DPDFLDIVALHR (16)	L2	1.198 (HLA-A*01:01)			1.020	0.825	0.940	98.57%	67.72%	82.89	207
DSDFMDIIRLHR (18)	L2	1.031 (HLA-B*40:01)			0.856	0.651	0.991	98.57%	67.23%	86.23	201.7

**Table 15:** Physicochemical properties of L1 and L2 DNA vaccine constructs.

construct	Epitope sequence (HPV-Type)	Antigenicity*	Molecular weight (Da)	Instability index of constructs	Solubility index of constructs**	*In vivo* half time
L1	EATVYLPPVPVSKVVA (16)	0.5397	28227	47.52(stable)	0.648(soluble)	>20 hours
	DNTVYLPPPSVARV (18)	0.2280				
	DTYRFVTSQAIACQK (16)	0.1995				
	DTYRFVQSVAITCQK (18)	0.3133				
	YLPPVPVSKV (16)	1.0876				
	YLPPPSVARV (18)	0.4104				
	DQFPLGRKFLL (16)	0.2797				
	DQYPLGRKFLV (18)	0.6476				
L2	PDFLDIVALHRPALTSR (16)	1.1115	25674	34.01(Stable)	0.407(soluble)	>20 hours
	SDFMDIIRLHRPALTSR (18)	0.4570				
	FFGGLGIGTGSGTGGR (16)	1.0724				
	DPSIVTLIEDSSVVTSGAP (18)	0.4474				
	KRASATQLYK (16)	0.1484				
	KRASVTDLYK (18)	0.4778				
	DPDFLDIVALHR (16)	1.6210				
	DSDFMDIIRLHR (18)	0.3371				

*higher rate shows high degree of peptide antigenicity.

**higher rate shows high degree of peptide solubility.

### Validation of the L1 and L2 DNA constructs

The designed HPV L1 and L2 genes were correctly cloned in pcDNA3.1 and pEGFP-N1 eukaryotic vectors. The presence of L1 and L2 genes were confirmed by digestion as a clear band of ~765 bp and ~700 bp on agarose gel for L1 and L2, respectively (data not shown). The recombinant endotoxin-free plasmids (*i.e*., pcDNA-L1 and pcDNA-L2) had a concentration range between 1.5 and 3.5 mg/mL.

### Evaluation of L1 and L2 DNA expression in HEK-293T cells

**Figure 3:**
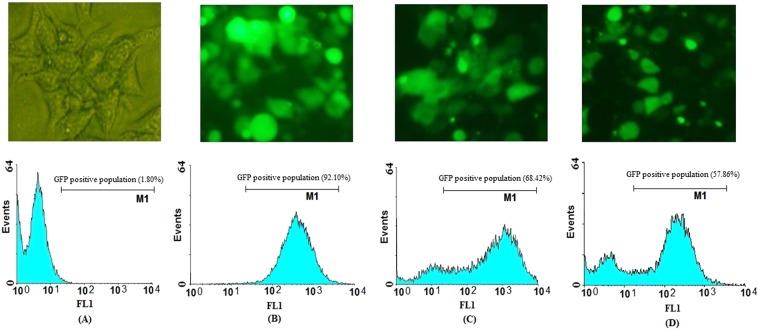
Evaluation of GFP (**B**), L2-GFP (**C**) and L1-GFP (**D**) DNA delivery into HEK-293T non-cancerous cells using TurboFect. Transfection efficiency was monitored by fluorescent microscopy (above) and flow cytometry (bottom) at 48 h post-transfection as compared to the negative control (**A**).

**Figure 4:**
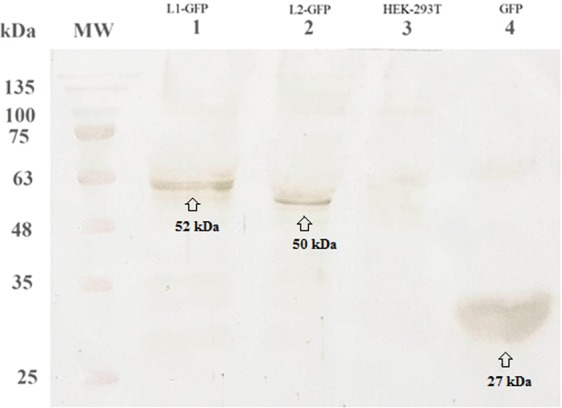
Identification of protein expression in HEK-293T cells using western blot analysis. The clear bands were observed for L1-GFP (lane 1, ∼52 kDa), L2-GFP (lane 2, ~50 kDa) and GFP (lane 4, ~27 kDa) proteins, respectively. Any clear band was not detected in un-transfected cells as a negative control (lane 3). MW is molecular weight marker (prestained protein ladder, 10–170 kDa, Fermentas).

*In vitro* DNA delivery of L1 and L2 into the eukaryotic cell line (HEK-293T) was performed by TurboFect as a transfection reagent. The levels of DNA expression were evaluated using fluorescence microscopy and flow cytometry at 48 h post-transfection. The data indicated that pEGFP-L1 and pEGFP-L2 can effectively penetrate into HEK-293T cells *in vitro*. The cellular uptake of the L1 and L2 genes into the HEK-293T cells was ~57.86% and ~68.42%, respectively. The delivery of pEGFP-N1 as a positive control was detected in approximately ~92.10% of HEK-293T cells (Fig. 3). Moreover, the spreading green regions were observed for L1 and L2 DNA delivery using TurboFect carrier by fluorescent microscopy in HEK-293T cells. On the other hand, western blot analysis indicated the successful expression of L1 and L2 proteins fused to GFP (*i.e*., L1-GFP and L2-GFP) using anti-GFP antibody. The data indicated the clear bands of ~52, ~50 and ~27 *kDa* for L1-GFP, L2-GFP and GFP, respectively using DAB substrate (Fig. 4).

### Measurement of tumor growth

**Figure 5:**
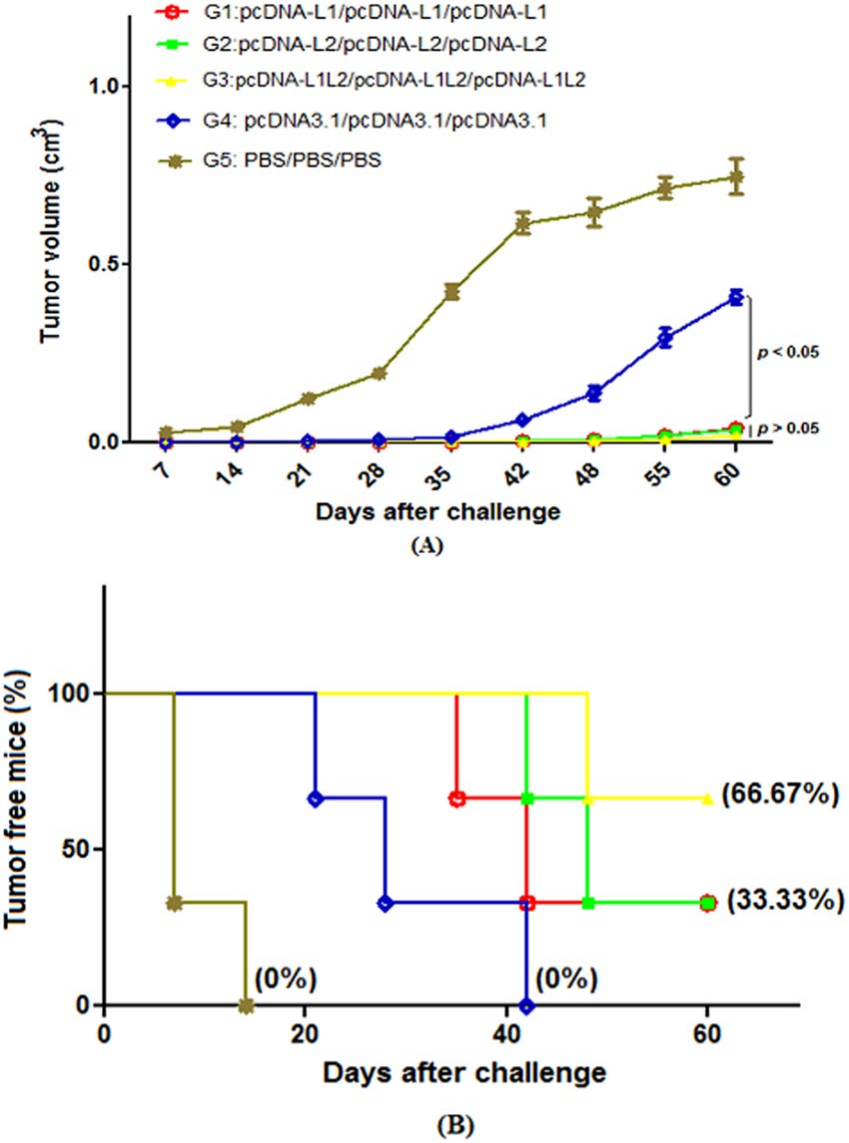
Tumor growth curve and survival percentage in different groups: The mice were challenged with 1 × 10^5^ C3 tumor cells two weeks after the last immunization: Tumor volumes were measured twice a week (**A**), the percentage of tumor-free mice (or survival rate) was evaluated in different groups (**B**)

To evaluate the prophylactic effects of the designed L1 and L2 DNA constructs, tumor growth and survival percentage were assessed in all groups for 60 days after challenging with C3 tumor cells. As shown in Fig. 5A, all test groups immunized with DNA constructs (G1, G2 & G3) demonstrated significantly lower tumor growth than that in control groups (PBS and empty vector, G4 & G5, *p* < 0.05). Our data showed progressive tumor growth in control groups on approximately 7–21 days (survival rate or tumor-free mice percentage: 0%). It was interesting that groups vaccinated with L1 DNA, L2 DNA and L1 + L2 DNA constructs similarly reduced the tumor growth (*p* > 0.05). As shown in Fig. 5B, group vaccinated with the mixture of L1 + L2 DNA constructs showed a higher survival rate (G3, ~66.67%) than L1 and L2 DNA constructs, alone (G1 & G2, ~33.33%).

### Antibody assay

**Figure 6:**
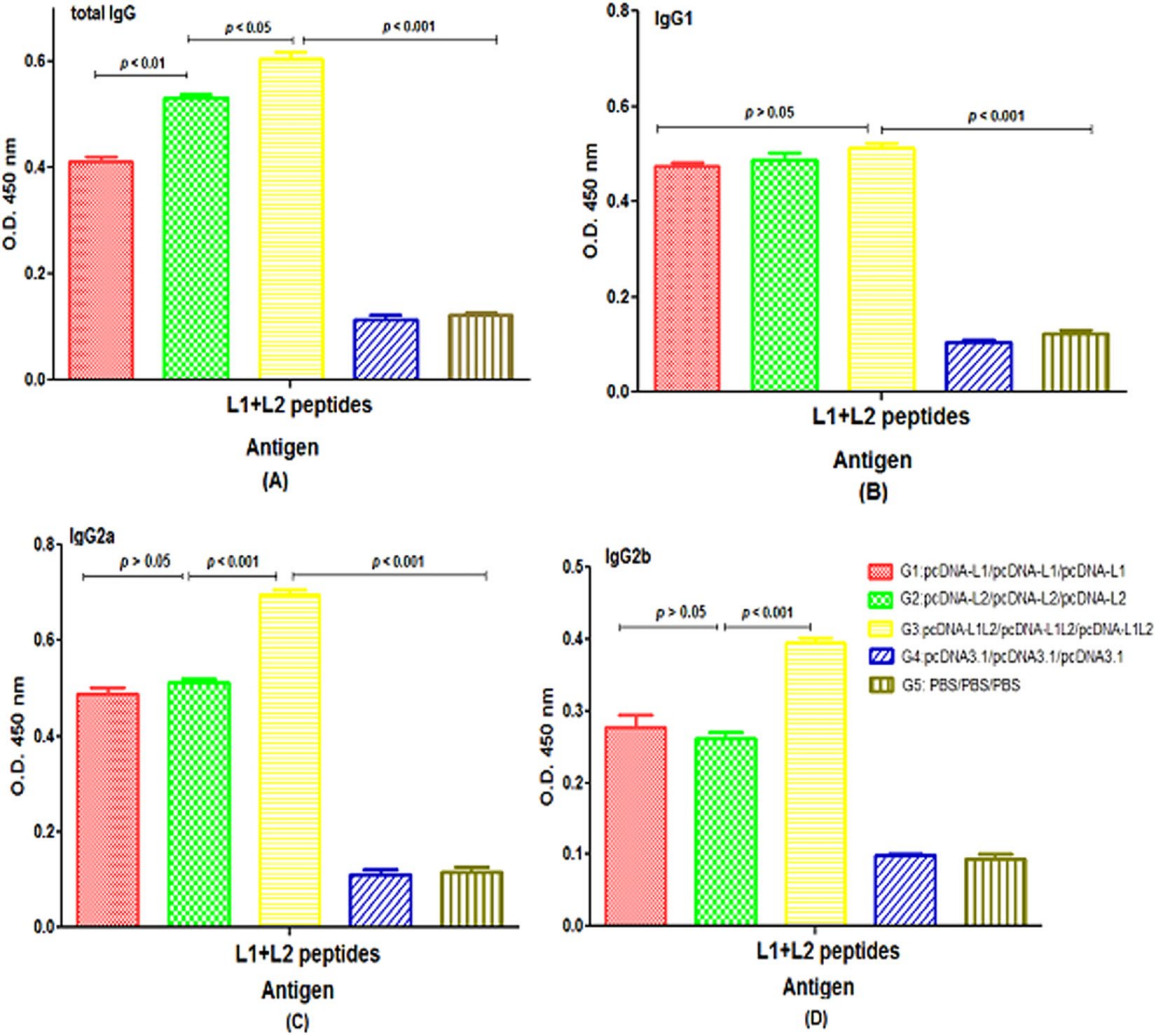
Antibody responses against the mixture of L1 + L2 peptides as an antigen in different groups: (**A**) total IgG, (**B**) IgG1, (**C**) IgG2a and (**D**) IgG2b; Mice sera were prepared from the whole blood samples of each group (n = 6) two weeks after the last immunization. All analyses were performed in duplicate for each sample shown as mean absorbance at 450 nm ± SD.

The levels of total immunoglobulin G (IgG), IgG2a and IgG2b in mice immunized with the mixture of L1 + L2 DNA constructs (G3) were significantly higher than other groups (*p* < 0.05, Fig. 6A,C,D). Moreover, our data showed that the levels of IgG1 were similar in all groups vaccinated with DNA constructs (G1, G2 & G3, *p* > 0.05, Fig. 6B). There are no significant differences in the secretion of IgG2a and IgG2b isotypes between groups receiving the L1 and L2 DNA constructs, alone (G1 & G2, *p* > 0.05, Fig. 6C,D). No significant anti-(L1 + L2) antibody responses could be detected in the sera of control groups, thus, the seroreactivities were completely L1 + L2 antigen-specific responses in mice.

### Cytokine assay

**Figure 7:**
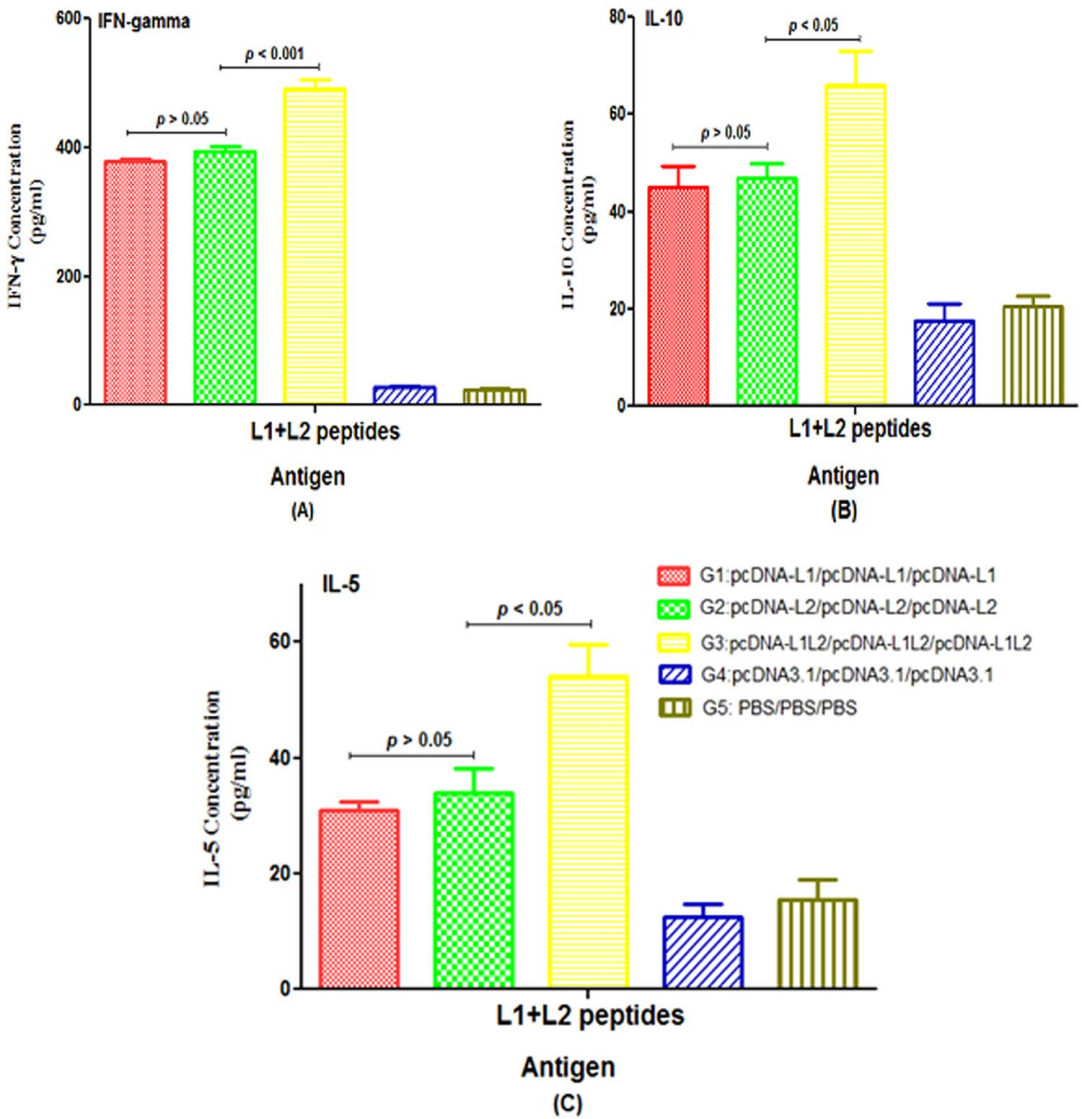
The levels of IFN-γ (**A**), IL-10 (B) and IL-5 (**C**) in vaccinated groups with different formulations: The pooled splenocytes were prepared from three mice in each group (n = 3 per group) and re-stimulated with the mixture of L1 + L2 peptides *in vitro*. The levels of cytokines were measured in the supernatant with ELISA as mean absorbance at 450 nm ± SD for each sample. All analyses were performed in duplicate for each sample.

The results of cytokine assay in each group showed that the levels of (L1 + L2)-specific IFN-γ, IL-10 and IL-5 secretions in groups immunized with L1 (G1), L2 (G2) and L1 + L2 (G3) DNA constructs were significantly higher than control groups (*p* < 0.05, Fig. 7). In contrast, there was no significant difference between mice vaccinated with L1 and L2 DNA constructs, alone (G1 & G2) for secretion of IFN-γ, IL-5 and IL-10 cytokines (*p* > 0.05). Among all the test groups, the group immunized with the L1 + L2 DNA construct (G3) showed the significant IFN-gamma, IL-5 and IL-10 responses compared to other groups (G1 & G2, *p* < 0.05, Fig. 7). Furthermore, our data indicated that the ratios of IFN-γ/IL-10 and IFN-γ/IL-5 were higher in all test groups as compared to control groups; therefore, they could trigger Th1 immune response.

### Granzyme B secretion

**Figure 8:**
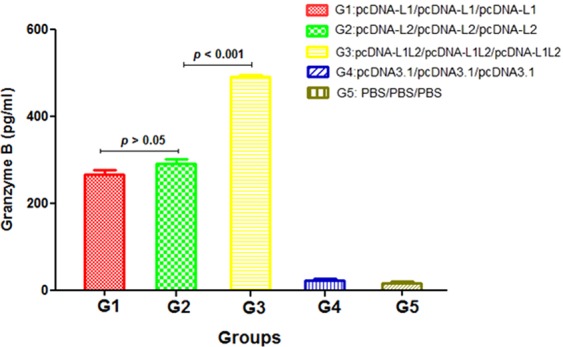
Granzyme B concentration measured by ELISA using the pooled splenocytes from three mice in each group (n = 3 per group). All analyses were performed in triplicate for each sample.

The secretion of Granzyme B in all test groups was significantly higher than the control groups (*p* < 0.05, Fig. 8). The group immunized with the L1 + L2 DNA construct (G3) produced significantly higher concentrations of Granzyme B than other groups (G1 & G2, *p* < 0.001). The level of Granzyme B in group receiving L1 DNA construct was similar to that in group receiving L2 DNA construct (*p* > 0.05).

## Discussion

In recent years, development of bioinformatics tools applied in vaccine researches could potentially save time and resources. Indeed, the immunoinformatics tools help to identify antigenic domains for designing a multi-epitope vaccine. With sequence-based technology advancement, now we have enough information about the genomics and proteomics of different viruses^22^. Thus, using various bioinformatics tools, we can design peptide vaccines based on a neutralizing epitope. For example, *in silico* design of an epitope-based vaccine against human immunodeficiency virus^23,24^, coronavirus^25^, dengue virus^26^, and Saint Louis encephalitis virus^27^ has already been reported.

While around 13 high-risk HPVs were recognized, current vaccines just protect humans from few types. An important limitation of the current vaccines is their narrow coverage. The accessibility of fully sequenced proteome from high-risk HPV strains provides a prospect for *in silico* screening of reliable peptide-based therapeutic vaccine candidates among billions of possible immunogenic peptides. *In silico* approaches are intended to reflect the possibilities for overcoming the above-mentioned difficulties in HPV multi-type vaccine. Gupta and coworkers designed prophylactic multiepitopic DNA vaccine using all the consensus epitopic sequences of HPVs L2 capsid protein. They also evaluated how engineering CpG motifs by bioinformatics tools could increase immunogenicity of DNA vaccines^28^. Hosseini *et al*. applied *in silico* analysis of L1 and L2 protein of HPV 11,16,18,31 and 45 types to identify universal peptide vaccine in order to protect against mentioned types^29^. In 2016, Singh *et al*. analyzed E1, E2, E6 and E7 proteins of high-risk HPV types to identify CD8^+^ T-cell epitopes. They suggested a pool of 14 peptides (9 to 43 amino acids) to provide the protection against high-risk HPV types^30^. Panahi and colleagues used a two-step method (consist of molecular docking and sequence-based approach) to determine immunogenic epitopes for induction of immune system against the oncoproteins of HPV 16, 18, 31 and 45 types^31^. In 2016, Wang and coworkers suggested the regions 51–58, 87–97, 214–220, 290–296, 335–341, 351–366, 408–418, 430–442 and 475–496 as putative B-cell epitopes for HPV16 L1 protein^32^. Sabah *et al*. used *in silico* immunoinformatics tools and reported a conserved 9 mer epitope (ESTVHEIEL) among all HPV58 types^33^. Bristo *et al*. designed MHC-I/MHC-II hybrid ras oncopeptide that could elicit T-cell reponse in an animal model^34^.

In this research, we designed a framework for the comprehensive analysis of L1 and L2 conserved regions of high-risk HPV types containing both MHC-I and MHC-II epitopes. The framework begins with conservancy analysis of all 13 high-risk HPV strains following with (1) B-cell epitope mapping, (2) T-cell epitope mapping (CD4^+^ and CD8^+^), (3) allergenicity assessment, (4) tap transport and proteasomal cleavage, (5) population coverage, (6) global and template-based docking and (7) data collection, analysis, and design of the L1 and L2 DNA constructs. For experimental analysis, the final L1 or L2 DNA constructs were cloned into mammalian expression vector with green fluorescent tag *(pEGFP* vector) and their expression was evaluated in the eukaryotic cells using *flow cytometry*, fluorescent microscopy and western blotting. Moreover, the L1/L2-specific antibody and T-cell immune responses induced by L1 and L2 DNA constructs were assessed in mouse tumor model.

At first, L1 and L2 sequences obtained from high-risk HPV types were aligned using MUSCLE algorithms. Conservancy analysis showed that five regions of HPV16,18 L1 protein (8–22, 95–132, 307–342, 398–425 and 449–473) and four regions of HPV16,18 L2 protein (11–40, 54–76, 96–120 and 278–305) were more conserved among other subtypes and could be analyzed as an immunoinformatics input. In B-cell epitope prediction, L1^8–22^, L1^408–421^, L1^404–417^, L2^22–35^, L2^100–113^, L2^94–107^ and L2^57–70^ had the highest epitope prediction scores. Unfortunately, a reliable method for prediction of B-cell epitope has not been revealed up to now and the sensitivity and specificity of existing methods were very low (the specificity and sensitivity of this method were 0.57 and 0.58, respectively). In the case of T-cell epitope prediction, *in silico* analysis has been significantly improved, thus, the results are more reliable. In this study, for MHC-I epitopes, L1^12–21^ (YLPPVPVSKV-type16 and YLPPPSVARV-type18), L1^460–470^ (DQFPLGRKFLL-type16), L1^461–471^ (DQYPLGRKFLV-type18), L2^11–20^ (KRASATQLYK-type16 and KRASVTDLYK-type18), L2^280–291^ (DPDFLDIVALHR-type16) and L2^273–284^ (DSDFMDIIRLHR-type18) epitopes had the highest binding affinity scores. In addition, above-mentioned epitopes had the highest T-cell epitope prediction scores which were obtained from proteasomal cleavage and tap transport analysis. High degree of conservancy was observed between subtypes for these epitopes (Table 4) especially in L1^460–470^ (DQFPLGRKFLL-type16) and L2^280–291^ (DPDFLDIVALHR-type18).

L1^460–470^ sequences were identical with HPV 16, 31, 33, 35, 39, 52, 58, 59, 68 types and had high similarity rate with HPV 51, 56, 18 and 45. In addition, L2^280–291^ sequences had higher degree of conservancy with HPV 16, 31, 33, 35, 51, 52, 58, and 56. For MHC-II prediction, L1^8–22^ (EATVYLPPVPVSKVV-type16), L1^416–430^ (DTYRFVTSQAIACQK-type16), L1^417–431^ (DTYRFVQSVAITCQK-type18), L2^281–297^ (PDFLDIVALHRPALTSR-type16), L2^274–290^ (SDFMDIIRLHRPALTSR-type18) and L2^54–69^ (FFGGLGIGTGSGTGGR-type16) epitopes had the highest binding affinity scores. Among them, L2^54–69^ had the greatest degree of conservancy (high similarity with all of the high-risk HPV types). One of the remarkable points is that L1^8–22^ and L2^57–70^ epitopes are the same (or overlapping with little difference (among B-cell and MHC-II selected epitopes. Due to a limitation of MHC-peptide binding prediction such as the gap between the peptides that are predicted to bind to MHC and those that experimentally bind^35^, flexible molecular docking has been employed to address this problem and raise the accuracy of MHC-peptide prediction. In the current study, template-based docking and also global docking were performed on the selected peptides to determine which peptide would get into the groove of MHC with the highest modeling scores. For MHC-I epitope, L1^12–21^, L1^460–470^ and L2^280–291^ sequences had the highest interaction similarity and cluster density scores. For MHC-II epitopes, L1^95–111^, L1^417–431^, L2^100–118^ and L2^281–297^ sequences had the highest docking scores. In this study, MHC-I-peptide docking scores confirmed MHC-I-peptide binding affinity scores because the same epitopes had the highest scores in both methods but in MHC-II molecular docking, the results were slightly different. One of the reasons is the significant conformational changes during the process due to the longer epitope length. As a general rule: the longer the length of the query peptide, the more torsions and conformational flexibilities^36^. Herein, due to longer peptide sequences, docking results in MHC-II were less accurate than MHC-I. For example, average similarity score in MHC-I was variable (171.8–259.7), but in MHC-II was 115.4–136. After the completion of the analysis and according to all of the above-mentioned parameters, two separate constructs were designed. In addition, accumulative population coverage of helper T-cell and CTL epitopes for the designed constructs were estimated. For the L1 and L2 constructs, the combination of 8 epitope candidates for helper T-cell and CTL in a single universal vaccine could involve all world population by the rate of 95.55% and 96.33%, respectively (Fig. 3). In previous studies, YLPPVPVSKV (HPV16 L1)^37^ and KRASVTDLYK (HPV18 L2)^21^ have been reported as potentially immunogenic epitopes. The ability of *in vitro* expression of the designed L1 and L2 DNA constructs was determined in HEK-293T cells using flow cytometry and western blot analysis. The transfection efficiency of the L1 and L2 DNA constructs was ~57.86% and ~68.42%, respectively indicating their high potency for delivery into the eukaryotic cells. As known, the use of a polytope DNA vaccine containing multiple T-cell and B-cell epitopes is an attractive strategy for developing a therapeutic and prophylactic vaccine against HPV infections. After *in vitro* assay, immunological experiments were performed in mice to determine the efficiency of the designed L1 and L2 DNA constructs without the use of adjuvant or delivery system for vaccine development. Similarly, some studies used the pcDNA vector harboring the gene of interest for immunization without any adjuvant^38,39^. Our data indicated that the groups immunized with L1, L2 and L1 + L2 DNA constructs increased antibody and T-cell responses as compared to control groups. Furthermore, the (L1 + L2)-specific immunity in mice receiving the mixture of L1 + L2 DNA constructs (G3) resulted in higher secretion of total IgG, IgG2a, IgG2b, IFN-γ, IL-5 and IL-10 cytokines as well as Granzyme B than other groups. The higher levels of IgG2a and IgG2b as well as IFN-gamma (as a Th1 cytokine) in this group drive T-cell responses toward Th1-type immunity. The studies showed that immunoglobulin G1 (IgG1) is related to a Th2-type response, while a Th1 response is associated with the induction of IgG2a and IgG2b in mice^40^. Regarding to our observations in protective studies, this regimen (L1 + L2 DNA construct: G3) could confer further protection against C3 tumor-challenged mice (survival rate: ~66.67%) depending on stimulation of CD4^+^ T cell-dominated Th1 responses as well as Granzyme B secretion (indicating CTL activity) as compared to the L1 or L2 DNA constructs, alone (survival rate: ~33.33%). These data showed high potency of the combined L1 + L2 DNA constructs versus each DNA construct alone as a prophylactic HPV vaccine. Taken together, immunoinformatics approaches have been emerged as a critical field for accelerating immunological researches. Yet, the immunoinformatics techniques applied to T-cells have more advancement than those dealing with B-cells^30^. Moreover, recently, due to the limited options for choosing an adjuvant in clinical trials, bioinformatics analyses have been developed to predict the best adjuvant. In this way, *in silico* studies help researchers saving time and resources, and also can guide the experimental work with higher probabilities of finding the desired solutions and with fewer trial and error repeats of assays. The accessibility of HPV genomic sequences and functional characterization of the genes involved in the virulence has significantly improved our understanding of the molecular foundation for the pathogenesis of HPV and offered a wealth of data that can be used to design new plans for vaccine design. Nowadays, powerful immune system simulators have been developed using bioinformatics tools which predict artificial immunity provided by the vaccine. These approaches could predict the best adjuvant for using in human vaccine studies. There is a multi-scale computational infrastructure approach which can stimulate the dynamics of the immune response induced by several vaccination formulations and predict optimal combination in terms of adjuvant type, dosage and timing. NetLogo is an agent-based modeling of the immune system running different simulations with different parameter settings. It also can interact with different modeling strategies including the investigation of pathogen growth, life cycle modeling environment for simulation complex phenomena^41–43^. Therefore, using these methods can increase efficiency and reduce costs in vaccine studies. In this study, for the first time, comprehensively integrated methods (using sequence-based tools in combination with flexible peptide-protein docking) were used to design highly immunogenic and protective vaccine candidates which were able to boost both humoral and cellular immune responses against all high-risk HPV types. In addition, *in vivo* analysis demonstrated high potency of the designed L1 and L2 constructs as combined in DNA-based vaccines without the use of adjuvant or delivery system. However, we will improve the efficiency of these DNA-based vaccines using a delivery system and also will compare their efficacy with the designed peptide-based vaccines along with adjuvants in near Future.

## Methods

### *In silico* analysis

#### Protein sequences retrieval

The reference L1 and L2 protein sequences of 13 high-risk HPV strains [16 (GI: 333031.L1 and GI: 333031.L2), 18 (GI: 60975.L1 and GI: 60975.L2), 31 (GI: 333048.L1 and GI: 333048.L2), 33 (GI: 333049.L1 and GI: 333049.L2), 35 (GI: 396997.L1 and GI: 396997.L2), 39 (GI: 333245.L1 and GI: 333245.L2), 45 (GI: 397022.L1 and GI: 397022.L2), 51(GI: 333087.L1 and GI: 333087.L2), 52(GI: 397038.L1 and GI: 397038.L2), 56 (GI: 397053.L1 and GI: 397053.L2), 58 (GI: 222386.L1 and GI: 222386.L2), 59 (GI: 557236.L1 and GI: 557236.L2, and 68(GI: 71726685.L1 and GI: 71726685.L2)] were extracted from PaVE database (https://pave.niaid.nih.gov/) and used as input for future bioinformatics analysis.

#### Protein alignments and conservancy analysis

To determine conserved epitopes between different subtypes, L1 and L2 sequence datasets were first aligned using SnapGene software 4.2.2 (From GSL Biotech; available at snapgene.com). After protein alignments analysis using muscle algorithms, the conserved epitopes of each protein were selected for immune-bioinformatics analysis such as B- and T-cell epitope prediction. Also, to calculate the degree of variability and conservancy of each epitope, IEDB epitope conservancy tools (http://tools.immuneepitope.org/tools/conservancy/) were used.

#### Linear B-cell epitope prediction

A successful vaccine must elicit a strong T-cell and B-cell immune response, but above all, provide protection against the disease being targeted. Therefore, it is essential to show that constructed immunogens are able to induce protective cellular and humoral immunity. Since the antibodies are induced against linear B-cell epitopes, it would be very difficult to synthesize long peptides with the native protein conformation resembling for the induction of protective antibodies. However, optimal peptide-based vaccines should be presented in a desired secondary structure of peptides in order to induce a specific humoral response^41,42^. For the B-cell epitope prediction of conserved regions in L1 and L2 proteins, BepiPred-2.0 server (http://www.cbs.dtu.dk/services/BepiPred-2.0/) was employed. In this study, epitope threshold value was set as 0.5 (the specificity and sensitivity of this method are 0.57 and 0.58, respectively)^41^.

#### T-cell epitope prediction

*MHC-I epitope prediction*: The initial step on applying bioinformatics to vaccine researches is to assess potentially immunoprotective epitopes. T-cell epitopes presented by MHC molecules are typically in a linear form containing 12 to 20 amino acids. This fact facilitates accurate modeling for the interaction of ligands and T-cells^44^. Thus, the most selective step in the presentation of antigenic peptide to T-cell receptor (TCR) is the binding of the MHC molecule^45^. In this study, we tried to use three different algorithms including Artificial Neural Networks (NetMHCpan 4.0 server^43^ (http://www.cbs.dtu.dk/services/NetMHCpan/), Quantitative matrix (Propred I^43^ (http://crdd.osdd.net/raghava/propred1/) and Published motifs (syfpeithi server^46^ (http://www.syfpeithi.de) to predict high-potential T-cell epitopes. For NetMHCpan, percentile rank was set at 0.5% for strong binders and 2% for weak binders and for Propred I threshold was set at 4%.

*MHC-II epitope prediction*: For MHC class II, NetMHCIIpan 3.2 server^47^ (http://www.cbs.dtu.dk/services/NetMHCIIpan/) and ProPred^48^ (http://crdd.osdd.net/raghava/propred/) were employed to predict potential interaction of helper T-cell epitope peptides and MHC-II. In this case, the threshold for strong and weak binders was set at 2% and 10%, respectively.

#### Prediction of MHC-I peptide presentation pathway

Investigating the Tap transport and proteasomal cleavage as well as affinity prediction of binding is essential in MHC-I presentation pathway. In this study, we used NetCTL 1.2 server combined with Tap transport/proteasomal cleavage tools (http://www.cbs.dtu.dk/services/NetCTL/) to access the prediction of antigen processing through the MHC class I antigen presentation pathway. In this method, parameters of weight on the C-terminal cleavage, Tap transport efficiency, and epitope identification were set to default (0.15, 0.05 and 0.75, respectively)^49^.

### Population coverage

Since the response to T-cell epitopes is restricted by MHCs, the selection of epitopes with multiple HLA-binding increases population coverage in defined geographical regions where the peptide-based vaccine might be employed. The coverage rate of population for each epitope was computationally validated using the IEDB population coverage tool^50^ (/population/iedb_input). In this study, individual epitope and its binding to HLA alleles were analyzed, and different geographic areas were also selected.

### Allergenicity and cross-reactivity assessment

Since proteins are very important in inducing allergenic reactions, the prediction of potential allergenicity is an important item in the safety assessment especially in the field of genetically modified foods, therapeutics, bio-pharmaceuticals *etc*.^51^. The food and agriculture organization (FAO) and world health organization (WHO) protocol includes three terms to evaluate the allergenicity of proteins which are defined as following: the term sensitivity refers to correctly predicted allergens (%), whereas specificity refers to correctly predicted non-allergens (%), and also accuracy refers to the proportion of correctly predicted proteins^19^. The allergenicity of the epitopes was analyzed by the PA^3^P (http://lpa.saogabriel.unipampa.edu.br:8080/pa3p/pa3p/pa3p.jsp) using Allergen online (8aa and 80 wordmatch) and AFDS-motif algorithms based on amino acid composition. The specificity of these methods is 95.43% (8aa), 92.88% (80aa) and 88.1% (ADFS)^52^. To assess cross-reactivity between peptide and human proteome, top-ranked epitope were analyzed by peptide matching program (https://research.bioinformatics.udel.edu/peptidematch/index.jsp)^53^.

### Peptide-protein flexible Docking

Computational docking methods have been known as an important tool for drug design^54^. With the rapid development of peptide therapeutics in rational drug design, the use of new techniques such as protein-peptide docking is inevitable. In this study, two different algorithms (template-based docking and global docking) were performed by GalexyPepDock server^55^ (http://galaxy.seoklab.org/cgi-bin/submit.cgi?type=PEPDOCK) and CABS Dock server^56^ (http://biocomp.chem.uw.edu.pl/CABSdock). To estimate the formation of MHC-peptide complex, the GalaxyPepDock server effectively models the structural 3D peptide-protein complexes from input peptide and protein sequences using the structure database and energy-based optimization (Template-based Docking). CABS-Dock server performs Global docking procedure which at first explicit fully flexible docking simulation and then clustering-based scoring. Receptor flexibility was limited by default to small backbone fluctuation but could be increased to include selected receptor fragments^56,57^. This study presented an example of MHC-peptide docking performed by each individual epitope and available PDB file (Table 13) of HLA alleles, separately.

#### Physicochemical properties of the designed L1 and L2 constructs

Based on L1 and L2 top-ranked epitopes, two different constructs were designed. The physicochemical properties of top-ranked epitopes such as solubility, molecular weight, estimated half-time, instability index and antigenicity were determined by ProtParam (https://web.expasy.org/protparam/) tools^58^, VaxiJen^59^ (http://www.ddg-pharmfac.net/vaxijen/VaxiJen/VaxiJen.html) and Protein-Sol (https://protein-sol.manchester.ac.uk/) server^60^.

## Experimental studies

### Construction of the recombinant plasmids

After bioinformatics analysis, the selected peptides were assembled in two separated constructs (Fig. 2). The pUC57-L1 and pUC57-L2 constructs were synthesized by Biomatik Company. For *in vitro* experiments, the pUC57-L1 and pUC57-L2 vectors were digested by *Xho*I/*Hind*III, and the L1 and L2 genes were subcloned into *Xho*I/*Hind*III sites of pEGFP-N1 vector, individually (*i.e*., pEGFP-L1 and pEGFP-L2). All the recombinant vectors were transformed into *Escherichia coli (E. coli*) DH5α strain. After extraction of plasmids from single colonies using Mini-Kit (Qiagen), the presence of inserted L1 and L2 fragments was confirmed by digestion with restriction enzymes and sequencing. For *in vivo* immunological assessment, the pUC57-L1 and pUC57-L2 vectors were digested by *Bam*HI/*Hind*III and the L1 and L2 genes were subcloned into *Bam*HI/*Hind*III sites of pcDNA3.1 (-) vector containing cytomegalovirus early promoter and enhancer sequence, individually (*i.e*., pcDNA-L1 and pcDNA-L2). Indeed, we used the pcDNA vector harboring CpG motif for *in vivo* studies. As a final point, the recombinant DNA vectors harboring L1 and L2 genes were purified by an endotoxin-free plasmid Extra EF kit (Macherey Nagel, Germany). The concentration and purity of the recombinant L1 and L2 DNA constructs were determined by NanoDrop spectrophotometry^61^.

### *In vitro* expression of L1 and L2 DNA constructs in HEK-293T cells

Human embryonic kidney cells (HEK-293T) were cultured in RPMI supplemented with 10% fetal bovine serum (FBS) at 37 °C and 5% CO_2_ atmosphere. After some passages, the cells were seeded in a 12-well plate. The optimal cell confluency for effective transfection was considered 70–80%. For the generation of TurboFect-plasmid DNA complex, 10 *μl* of TurboFect (Thermo Scientific) and 2 μg of each plasmid (pEGFP-L1, pEGFP-L2 and pEGFP-N1 as a positive control) were mixed and incubated for 15 min at room temperature. Then, the complex was added to each well in serum-free media. In addition, the non-transfected HEK-293T cells were used as negative control. After six hours, the media was replaced with the completed RPMI medium. Finally, the cells were harvested, washed and resuspended in PBS buffer, to analyze the expression of L1 and L2 DNA constructs using flow cytometry, fluorescent microscopy and western blotting at 48 hr after transfection^61^.

### Western blot analysis

HEK-293T cells were scraped from their plates and washed with PBS1X. After washing steps, the cells were lysed in whole-cell lysis buffer (10% glycerol, 1 nM DTT, 2 mM natrium fluoride, 0.2% Triton X-100, 0.5 EDTA in PBS pH = 7.4). The extracted protein samples (L1-GFP, L2-GFP and GFP) were separated by SDS-PAGE in 12.5% (w/v) polyacrylamide gel and transferred to nitrocellulose membrane (Millipore). The membrane was equilibrated with TBST (Tris-buffered saline Tween-20) solution containing 2.5% BSA (Bovine albumin serum) overnight. The anti-GFP polyclonal antibody (1:5000 v/v; Acris antibodies GmbH) was used to recognize the expressed proteins under standard procedures. The immunoreactive protein bands were visualized by detection of peroxidase activity using a substrate named as 3, 3′-diaminobenzidine (DAB, Sigma)^61^.

### Peptide constructs synthesis

For immunological assay (*i.e*., secretion of antibody, cytokine and Granzyme B), two peptide constructs (L1 and L2 peptides, Fig. 2) were synthesized by BioMatik Co. with more than 85% purity.

### Mice immunization

Five groups of six female C57BL/6 mice (obtained from the breeding stocks maintained at Pasteur Institute of Iran; MHC haplotype B/H-2Kb/H-2Db) were immunized on days 0, 14, and 28 (*i.e*., three times with a 2-week interval) with 50 µg of each plasmid DNA (pcDNA-L1 or pcDNA-L2: G1 or G2) or their combination (pcDNA-L1+ pcDNA-L2: G3) at the right footpad as shown in Table 16. The control groups (G4 and G5) received pcDNA3.1 and PBS, respectively. All mice were maintained under specific pathogen-free conditions^62^. Moreover, all of the animal experimental procedures were approved by Animal Care and Use Committee of Pasteur Institute of Iran and carried out according to the Animal Experimentation Regulations of Pasteur Institute of Iran (national guideline) for scientific purposes (code: 976).

**Table 16:** Immunization program for *in vivo* analysis.

Groups	First injection or priming (Day 0)	Second injection or first booster (Day 14)	Third injection or second booster (Day 28)	Challenge with C3 cells (Two weeks after second booster)
G1	pcDNA-L1	pcDNA-L1	pcDNA-L1	C3
G2	pcDNA-L2	pcDNA-L2	pcDNA-L2	C3
G3	pcDNA-L1 + pcDNA-L2	pcDNA-L1 + pcDNA-L2	pcDNA-L1 + pcDNA-L2	C3
G4 (Control)	pcDNA3.1 (empty vector)	pcDNA3.1 (empty vector)	pcDNA3.1 (empty vector)	C3
G5 (Control)	PBS	PBS	PBS	C3

### Monitoring tumor growth

For *in vivo* protection assay, vaccinated mice were subcutaneously challenged in the right flank with C3 tumor cells (1 × 10^5^ cells), two weeks after the last injection. The C3 tumor cells contain whole HPV16 genome, and the presence of L1 and L2 genes was confirmed in the previous studies^63^. Tumor growth and the percentage of tumor-free mice were monitored twice a week by palpation for 60 days post-challenge. At each time, tumor volume was calculated by this formula: V = (a^2^b)/2 (a = the smallest diameter and b = the biggest diameter)^62^.

### Antibody assay secreted from B-cells

Two weeks after the last injection, serum samples were collected from each group. The levels of goat anti-mouse immunoglobulin G1 (IgG1), IgG2a, IgG2b and total IgG antibodies (diluted 1:10,000 in 1% BSA/PBS-Tween, Sigma) secreted from B-cells were measured in the pooled sera of each group by indirect ELISA. The coated antigens were the mixture of L1 and L2 synthetic peptides (5 μg/mL). Moreover, mice sera were diluted 1:100 in 1% BSA/PBS-Tween^64^.

### Cytokine assay secreted from T-cells

Three mice from each group were sacrificed and the spleens were removed. The red blood cell-depleted pooled splenocytes (2 × 10^6^ cells/ml) were cultured in 48-well plates for 72 h in the presence of 5 μg/mL of L1 + L2 peptides, RPMI 5% as negative control and 5 μg/mL of concanavalin A (ConA) as positive control in complete RPMI culture medium. The supernatants were harvested to assess the secretion of IFN-γ, IL-5 and IL-10 from T-cells using the sandwich-based ELISA method (R&D Systems) according to the manufacturer’s instructions. All data were represented as mean ± SD for each sample^65^.

### Granzyme B assay (*in vitro* CTL activity)

To measure Granzyme B (GrB) by ELISA, the P815 target cells (T) were seeded into 96-well plates (2 × 10^4^ cells/well) incubated with the mixture of L1 and L2 peptides (~30 μg/mL) for 24 h. Then, the prepared splenocytes (Effector cells: E, before section) were counted and added to the target cells at E: T ratio of 100: 1 in complete RPMI culture medium for 6 h incubation. Finally, the supernatants were harvested to measure the concentration of GrB by ELISA (eBioscience kit) according to the manufacturer’s instruction^64^.

### Statistical analysis

Statistical analyses were performed by Prism 7.0 (GraphPad, San Diego, California, USA) to determine the differences between the control and test groups using one-way ANOVA and student’s *t*-test. Survival rate or the percentage of tumor-free mice was evaluated using the log-rank (Mantel-Cox) test. The value of *p* < 0.05 was considered statistically significant.
